# The biophysical nature of cells: potential cell behaviours revealed by analytical and computational studies of cell surface mechanics

**DOI:** 10.1186/s13628-015-0022-x

**Published:** 2015-05-12

**Authors:** Ramiro Magno, Verônica A Grieneisen, Athanasius FM Marée

**Affiliations:** Theoretical Biology/Bioinformatics, Dept. of Biology, Utrecht University, Padualaan 83584 CH, Utrecht, Netherlands; Computational and Systems Biology, John Innes Centre,, Norwich Research Park, NR4 7UH, Norwich, UK

**Keywords:** Cell surface mechanics, Cellular Potts model, Cell shape, Cortical tension, Membrane tension, Adhesion, Tissue packing, Cellular vertex-models, Grid anisotropy, 3D modelling, Cell sorting, Cellular dynamics

## Abstract

**Background:**

The biophysical characteristics of cells determine their shape in isolation and when packed within tissues. Cells can form regular or irregular epithelial structures, round up and form clusters, or deform and attach to substrates. The acquired shape of cells and tissues is a consequence of (i) internal cytoskeletal processes, such as actin polymerisation and cortical myosin contraction, (ii) adhesion molecules within the cell membrane that interact with substrates and neighbouring cells, and (iii) processes that regulate cell volume. Although these processes seem relatively simple, when combined they unleash a rich variety of cellular behaviour that is not readily understandable outside a theoretical framework.

**Methods:**

We perform a mathematical analysis of a commonly used class of model formalisms that describe cell surface mechanics using an energy-based approach. Predictions are then confirmed through comparison with the computational outcomes of a Vertex model and 2D and 3D simulations of the Cellular Potts model.

**Results:**

The analytical study reveals the complete possible spectrum of single cell behaviour and tissue packing in both 2D and 3D, by taking the typical core elements of cell surface mechanics into account: adhesion, cortical tension and volume conservation. We show that from an energy-based description, forces and tensions can be derived, as well as the prediction of cell behaviour and tissue packing, providing an intuitive and biologically relevant mapping between modelling parameters and experiments.

**Conclusions:**

The quantitative cellular behaviours and biological insights agree between the analytical study and the diverse computational model formalisms, including the Cellular Potts model. This illustrates the generality of energy-based approaches for cell surface mechanics and highlights how meaningful and quantitative comparisons between models can be established. Moreover, the mathematical analysis reveals direct links between known biophysical properties and specific parameter settings within the Cellular Potts model.

## Background

The assembly of a multicellular organism is achieved, alongside cell division, apoptosis and differentiation, by changing cell shape and tissue topology. Such cellular and tissue dynamics are highly determined by the cell’s physical properties. Therefore, to increase our understanding of morphogenesis, we need quantitative analyses and descriptions of the biomechanical properties of cells and tissues. However, despite the recent advances in this field [1-12], we still lack detailed knowledge as well as computational power to approach cell mechanics from a molecular perspective. Accordingly, there are several multicellular modelling approaches that waive the fine detail of specific molecular interactions by describing cell properties on a biophysical cellular level (see [13] for a review). Such approaches are analogous to the simplification of considering the surface tension of a liquid droplet, instead of explicitly specifying the molecular interactions involved. These cellular models maintain the desired mesoscopic cell features, while facilitating the comparison between simulations and experimental data, given its reduced number of variables and effective parameters [14-16]. Although such mesoscopic approaches lack direct links between the molecular level and the macroscopic biological outcome, they remain powerful instruments for comprehending cellular behaviour, since they allow the investigation of novel regulatory elements and biochemical processes. Lecuit and Lenne [17] evaluated an overarching class of such mesoscopic biophysical models, the Cell Surface Mechanics (CSM) models. These describe how biologically-generated tensions – namely adhesion-derived tension, cortical tension and tension due to the cell’s internal pressure – affect (i) the displacement of the cell’s membrane, (ii) modify the cell’s shape and (iii) influence its dynamics. Nevertheless, major issues prevent us from gaining optimal understanding from these models.

Firstly, it is not always clear how to quantitatively link experimental observations to model implementations. We will therefore discuss how biophysical properties are linked to subcellular components, such as the structure and mechanics of the cytoskeleton and the interactions between adhesion molecules among neighbouring cells. On a pragmatic level, this leads us to derive a protocol for CSM class simulations [17], to determine and modify expected cell behaviour in simulations and help establish a link with experimental observations.

A second source of confusion relates to the wide variety of computational implementations used to describe tissues. It is not yet evident how a model-to-model comparison can be drawn, confusing the biology community when trying to compare common insights and features between different theoretical studies. Likewise, modellers are frequently not aware of the common features and artefacts of their implementations, making them susceptible to overlooking valuable information from parallel studies. We address this second concern in two ways: (i) we initially discuss in detail how a commonly used lattice-based model formalism – the Cellular Potts model (CPM) – can (and should) be seen as being part of the CSM class, and how to determine its parameters and expected dynamics, whilst ensuring appropriate scaling; and (ii) we compare CPM simulations to analytical predictions as well as other CSM implementations, and highlight overlaps and interesting deviations.

We use the CPM as a reference model formalism from the CSM class. Originally, CPM was proposed to describe cellular dynamics based on differential adhesion, thereby defining tissue surface tension relations [18,19], to consolidate Steinberg’s Differential Adhesion Hypothesis (DAH) [20]. Classical CPM simulations, in which cells display an internal pressure and positive adhesion coupling constants, successfully captured cell sorting phenomena [18,19,21]. However, in the classical formulation the CPM did not include the effects of cortical tension. Ouchi et al. [22] have proposed an extension with a term representing the surface constraint tension (to modify the relationship between cell motility within aggregates and adhesion strength [23]), alongside using negative instead of positive coupling constants for the adhesion (discussed later). Recently, such a surface constraint term has been identified as the cortical tension, the third “ingredient” underlying cell surface mechanics [17,24]. Since then, cortical tension within the extended CPM has been used in several multicellular modelling studies, for example, in the description of cortical-tension dependent cell shape alterations in dendritic cells [14]; in reverse engineering of the critical parameters determining Drosophila’s eye geometry [24]; and to understand germ-layer organisation in zebrafish [15]. A number of other computational model formalisms have been proposed which, analogous to the CPM, use an energy-based description for volume conservation, adhesion and cortical tension [25-28]. They adopt, however, different ways of describing the cell shape as well as its dynamics. Such models have been used to explore the role of the interfacial tension, reproducing, through parameter changes of the CSM, different forms of epithelial cell packing [25]; plausible mechanisms underlying cell intercalation [27]; and again, the Drosophila’s eye geometry [26].

Surprisingly, despite the growing acceptance of the concept and importance of cortical tension from both modelling and cell biology communities, a clear explanation for the role of cortical tension in cell surface mechanics is still lacking. In fact, although several analytical studies regarding CSM have been published [29,30], it is still unclear what cell behaviour can be expected even when just a single cell is considered in terms of its cortical tension. For multicellular modelling, such a baseline understanding is vital to pinpoint the role of each biophysical parameter, allowing us to distinguish and predict beforehand individual- and collective-cell-driven mechanisms. We used the CPM formalism to numerically verify our analytical predictions and to determine their implications in actual cell dynamics, highlighting the fact that the analytical results are independent of the specific simulation framework.

## Methods

### The cell surface mechanics model

**Figure 1:**
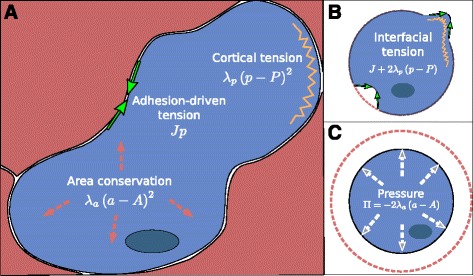
Cell surface mechanics.**(A)** Illustration of the three main forces regulating the cell surface mechanics: adhesion-driven tension, cortical tension and pressure. Adhesion is described as a constant tension *J* along the surface (green arrows). The cortical tension is described by an elastic tension with equilibrium length *P* and elasticity constant of *λ*_*p*_ (orange spring). **(B)** The interfacial tension is defined as the altogether adhesion-driven and cortical tensions. **(C)** Deformations away from the cell’s target area *A* generates a pressure *Π* (white arrows).

From empirical evidence, it is well known that cells maintain and regulate their size through osmotic pressure control [31]; that internal cellular cortical regulation, through myosin contraction, deforms cells; and that interactions with other cells influence individual shapes, since when two cells are brought together to adhere, their contact interface will extend dependent on their adhesion strengths [32]. Energy-based models for cell surface mechanics bring these three main observations together through a description of three independent energy-terms (Figure 1A). Pressure is described by the energy variation due to volume deviation relative to the cell’s target volume (resting volume). The contractility driven by the cell cortex actin-myosin complex is described by an elastic (Hookean) tension applied to its membrane, i.e. the energy increases quadratically with the deviation from the membrane’s resting length. The adhesion between two interfaces, such as cell-cell or cell-extracellular contacts, can be simply described as a constant surface tension term. An in-depth comparison how and to which extent those biophysical processes can be linked to the underlying cell biology can be found in [17]. Although the effective surface tension – interfacial tension – that follows from these terms is an equilibrium property, it also proves to be a powerful concept in understanding the dynamics of cells and tissues [17,33]. Most studies until now considered 2D epithelial cell layers, although some work has been done on 3D tissue (e.g. [14]). Because in epithelial tissue cell adhesion and cell-cell contacts are typically restricted to a small zone close to the apical cell surface, it is generally considered reasonable to treat epithelial cell packing as a 2D problem [24,25].

Although the nomenclature varies throughout the literature, in all 2D studies mentioned above the energy function *E* takes the form of

(1) $$ E\left(p,a\right|J,\lambda_{p},P,\lambda_{a},A)=Jp+\lambda_{p}\left(p-P\right)^{2}+\lambda_{a}\left(a-A\right)^{2}\,,  $$

where *p* and *a* are the perimeter and area of the cell (see Figure 1A). The function uses five parameters for the cellular properties: *J*, an energy per contact length due to adhesion to other cells or the surrounding medium; *P*, the membrane resting length; and *A*, the target cell area (resting area); while the constraints are modulated by the Lagrange multipliers *λ*_*p*_ and *λ*_*a*_ (comparable to elastic constants), which weigh the relative tension contributions of actin-myosin contraction and cell deformations, respectively. Although modifications of the above energy function could and have been proposed (see, e.g., [34]), almost all studies on CSM have been using this basic framework, sometimes further simplified (see, e.g., [19,25]), or extended with additional terms that, for example, capture chemotaxis, the microstructure of the extracellular matrix or fluid dynamics [35-37]. These extensions, such as combining CSM with chemotaxis, can trigger highly intricate and sophisticated dynamics [38]. Nevertheless, understanding the dynamics of the core CSM model is an essential ground step to enable understanding of the full process and in interpreting the meaning and consequences of any subsequent model extension.

Note that the above equation is a simplification which assumes that the cell is completely surrounded by homogeneous contacts (which could be other cells or medium). In the case of an heterogeneous cell environment, the first term, in its most general form, should be written as $\sum _{\sigma '}J_{\sigma '}p_{\sigma '}$, where *σ*^′^ indicates surrounding medium and/or neighbouring cells; $J_{\sigma ^{\prime }}$ the energy per contact length specifically for the interface between the cell and *σ*^′^; and $p_{\sigma ^{\prime }}$ the length of that given interface. The sum over all of the cell interfaces’ lengths gives its perimeter $p=\sum _{\sigma '}p_{\sigma '}$. For the initial analysis we will assume the environment to be homogeneous. There has been ample discussion whether it is more reasonable to use positive or negative *J*-values [22,24]. Part of the issue is that *J*-values may be used to encode several biological processes, making it a challenge to quantify or ascribe to them a biological meaning (see [29] for a detailed discussion). We therefore assume that its sign (as well as the sign of *P*, see discussion on mapping between *J* and *P* below) is undetermined. It is nonsensical, however, to consider negative values for the perimeter and area constraints, and it seems unreasonable to use a negative target area. Moreover, while in many modelling studies no perimeter constraint is being used (corresponding to *λ*_*p*_=0), it is nonsensical to have no area constraint. We therefore assume that *λ*_*p*_ and *A* are always non-negative and *λ*_*a*_ is positive. We initially focus on a 2D cell, and later extend our analysis to 3D tissues.

Note that the formalism, besides discarding any intracellular detail, also describes cell surfaces without explicit “surface elements”, whose movement could be followed over time and would require energy to move closer/away from each other (when not affecting its perimeter or area). While clearly being a coarse simplification, this reduced level of membrane complexity is what allows CSM models to capture complex tissue dynamics involving many cells. (Note that while numerically CSM dynamics might be calculated through displacements of introduced surface elements, they are not relevant for the energy calculation of the configuration, and hence for the dynamics itself.)

From the energy function above, we can derive important quantities that will greatly facilitate the understanding of cell and tissue dynamics. Firstly, the cell’s interfacial tension *γ* — the work required to extend the membrane by a unit area — is expressed in 2D as the change in energy per unit perimeter length (Figure 1B) and depends on both the adhesion and the cortical tension,

(2) $$ \gamma=\frac{\partial E}{\partial p}=J+2\lambda_{p}\left(p-P\right)\,.  $$

We want to emphasise that while both forces contribute to the cell interfacial tension, only adhesion is specific to the cell-cell or cell-medium interactions at the interface. Also, although on a regulatory level adhesion and cortical tension might be biologically linked, they can be physically separated within this energy description. The interfacial tension can be split up into a length-independent component, *J*−2*λ*_*p*_*P*, and a component which depends linearly on the perimeter length, 2*λ*_*p*_*p*. We can thus write

(3) $$ \gamma=\tau+2\lambda_{p}p\,,  $$

where *τ* is defined as the length-independent component of the interfacial tension. The sign of *τ* is undetermined, while the length-dependent component is always non-negative.

The pressure *Π* within the cell that contributes to a force per unit membrane area can be represented as the work required per unit volume decrease or, equivalently, the decrease in energy per unit volume increase (in 2D, area increase) (Figure 1C):

(4) $$ \Pi=-\frac{\partial E}{\partial a}=-2\lambda_{a}\left(a-A\right)\,.  $$

The force $\vec {F}$ applied at a certain point of the cell’s membrane due to the above energy function (Eq. 1) is the negative of the local gradient of the function at that point, ∇*E*, which can be elegantly rewritten in terms of changing area and perimeter:

(5) $$ \vec{F}=-\nabla E=\Pi\nabla a-\gamma\nabla p\,.  $$

The force $\vec {F}$ represents a vector at a point $\vec {x}$ on the membrane, which can be decomposed into an interfacial tension force $\vec {F_{\gamma }}=-\gamma \nabla p$ and a pressure driven force $\vec {F_{\Pi }}=\Pi \nabla a$. An imbalance between these forces should lead to a dynamical change of the cell shape. It is important to note that biological cells are highly dissipative objects, for which viscous forces greatly exceed inertial forces (low Reynolds number, see [39]). Thus, the motion of cell deformations due to the force $\vec {F}$ acting on the cell membrane is usually assumed to be overdamped, with inertial terms being negligible when compared to dissipative terms, leading to first-order dynamics, i.e. $\vec {F}\propto d\vec {x}/dt$ [13,40].

As one of our aims is to clarify how model parameters determine cell and tissue behaviour, we will first characterize the equilibrium states of single cells. To do so, we use the above definitions and derived variables.

## Results

### Single cell analysis

To understand the role of the biophysical parameters on the cell mechanics we started asking two basic questions. Firstly, what are all the possible equilibrium cell sizes, what is their stability, and how are these defined by the parameters; and secondly, what is the parameter regime for which, at equilibrium, the cell’s interfacial tension (*γ*) is positive. The answer to the former question tells us for which cell size(s) the forces acting on the cell are in balance, while the answer to the latter question enables us to determine whether the cell shape is stable. In 2D, when *γ*>0 the cell tends to minimise its perimeter relative to the enclosed area *a*. Under this condition, the circle is the cellular shape that best minimises the *a*/*p* ratio [41].

We begin by assuming that the cell has a circular shape, as this is the minimal-energy solution given positive interfacial tension. In such a scenario, the cell’s perimeter and area cannot be independently specified. Instead both can be specified as a function of the cell radius *r*, with *p*=2*πr* and *a*=*πr*^2^, respectively. Given the circular shape assumption, the energy *E* can be expressed as a function of *r*, where *R*_*p*_=*P*/2*π* and $R_{a}=\sqrt {A/\pi }$ are the reciprocal target radii of *P* and *A*,

(6) $$ \begin{aligned} E\left(\left.r\right|J,\lambda_{p},R_{p},\lambda_{a},R_{a}\right)=&\,2\pi rJ+\lambda_{p}\left(2\pi r-2\pi R_{p}\right)^{2}\\&+\lambda_{a}\left(\pi r^{2}-\pi {R_{a}^{2}}\right)^{2}\,. \end{aligned}  $$

Once again, from this energy function we can extract the cell’s interfacial tension (*γ*) and pressure (*Π*), but now as a function of the radius. For the circular cell, Eq. 2, Eq. 4 read as

(7a) $$\begin{array}{*{20}l} \gamma & =J+4\pi\lambda_{p}\left(r-R_{p}\right)\,, \end{array} $$

(7b) $$\begin{array}{*{20}l} \Pi & =-2\pi\lambda_{a}\left(r^{2}-{R_{a}^{2}}\right)\,. \end{array} $$

#### Positive interfacial tension at the equilibrium cell radii

Under positive interfacial tension, energy reduction leads to surface minimization. When the interfacial tension is negative, the tendency of reducing the surface for a determined area is lost, as there is no energy cost to additional surface – in fact, the tendency is to increase the length of the interface, causing cells which are constrained in area to adopt different non-circular and ruffled shapes. Thus, to predict the qualitative behaviour of the cell regarding shape, it is therefore important to determine the sign of the interfacial tension at the equilibrium radius.

To determine the equilibrium cell radii, we first take the derivative of *E* with respect to the radius of the cell,

(8) $$ \frac{\partial E}{\partial r}=2\pi\left(J+4\pi\lambda_{p}\left(r-R_{p}\right)\right)+2\pi r\left(2\pi\lambda_{a}\left(r^{2}-{R_{a}^{2}}\right)\right)\,.  $$

By definition, at equilibrium the energy gradient is zero (provided *r*^∗^>0, because an equilibrium radius *r*^∗^<0 is nonsensical, and *r*^∗^=0 is always an equilibrium, independent of the energy gradient). Therefore, the equilibrium radius can be found by solving for $\frac {\partial E}{\partial r}=0$ (Eq. 8). To avoid directly analysing this cumbersome cubic equation, we rewrite Eq. 8 into a form from which we can directly determine the sign of the interfacial tension at equilibrium. The first step is to substitute Eq. 7 into Eq. 8, which yields

(9) $$ \frac{\partial E}{\partial r}=2\pi\left(\gamma(r)-r\Pi(r)\right)\,.  $$

Then we bring in the equilibrium radius *r*^∗^ (for which the energy gradient is zero) giving us

(10) $$ \left.\frac{\partial E}{\partial r}\right|_{r=r^{*}}=2\pi\left[\gamma\left(r^{*}\right)-r^{*}\Pi\left(r^{*}\right)\right]=0\,.  $$

For a cell to have a stable circular shape, the interfacial tension at the equilibrium radius has to be positive, i.e. *γ*(*r*^∗^)>0. From the requirement *γ*(*r*^∗^)>0 it follows that

(11a) $$\begin{array}{*{20}l} J+4\pi\lambda_{p}\left(r^{*}-R_{p}\right) & >0\,, \end{array} $$

(11b) $$\begin{array}{*{20}l} r^{*} & >\frac{-\tau}{4\pi\lambda_{p}}\,, \end{array} $$

where, as before, *τ* describes the perimeter-independent component of the interfacial tension, *τ*=*J*−4*πλ*_*p*_*R*_*p*_. Moreover, from Eq. 10 we find that at equilibrium *γ*(*r*^∗^)=*r*^∗^*Π*(*r*^∗^). The requirement *γ*(*r*^∗^)>0 therefore also implies that *r*^∗^*Π*(*r*^∗^)>0, and thus

(12a) $$\begin{array}{*{20}l} -2\pi\lambda_{a}r^{*}\left(r^{*2}-{R_{a}^{2}}\right) & >0\,, \end{array} $$

(12b) $$\begin{array}{*{20}l} r^{*} & <R_{a}\,, \end{array} $$

again provided *r*^∗^>0. By combining inequalities Eq. 11 and Eq. 12, the requirement for an equilibrium radius to have a positive interfacial tension becomes

(13) $$ \frac{-\tau}{4\pi\lambda_{p}}<r^{*}<R_{a}\,.  $$

Two conclusions can be drawn from this inequality. First, a solution is only possible when

(14a) $$\begin{array}{*{20}l} \frac{-\tau}{4\pi\lambda_{p}} & <R_{a}\,, \end{array} $$

(14b) $$\begin{array}{*{20}l} \tau & >-4\pi\lambda_{p}R_{a}\,. \end{array} $$

Consequently, the interfacial tension at *r*^∗^>0 is going to be negative for *anyτ*<−4*πλ*_*p*_*R*_*a*_, independently of the specific value of *r*^∗^. Secondly, when *τ*>−4*πλ*_*p*_*R*_*a*_, Eq. 13 is always fulfilled, and hence it follows that the interfacial tension at *r*^∗^ has to be positive. This can be concluded in the following way: When *τ*>−4*πλ*_*p*_*R*_*a*_, it is not immediately clear that the interfacial tension is positive, but it does directly imply that $\frac {-\tau }{4\pi \lambda _{p}}<R_{a}$. Only when $\frac {-\tau }{4\pi \lambda _{p}}<r^{*}<R_{a}$ the interfacial tension is positive, but there seem to be other possibilities which could give a negative interfacial tension, namely $r^{*}<\frac {-\tau }{4\pi \lambda _{p}}<R_{a}$ and $\frac {-\tau }{4\pi \lambda _{p}}<R_{a}<r^{*}$. Now let’s assume that the first situation applies, i.e. $r^{*}<\frac {-\tau }{4\pi \lambda _{p}}<R_{a}$. Because of $r^{*}<\frac {-\tau }{4\pi \lambda _{p}}$, we know from Eq. 11b that *γ*(*r*^∗^)<0. Consequently, to fulfil Eq. 10, *r*^∗^*Π*<0 as well. But this implies that (Eq. 12) *r*^∗^>*R*_*a*_, which renders this approach inconsistent. Using the inverse reasoning, it is clear that the other situation, i.e. *r*^∗^>*R*_*a*_, is also not possible. Therefore, when *τ*>−4*πλ*_*p*_*R*_*a*_, it automatically follows that $\frac {-\tau }{4\pi \lambda _{p}}<r^{*}<R_{a}$, and so the interfacial tension at *r*^∗^ is always positive. This allows for a straightforward calculation to determine if the interfacial tension at *r*^∗^ is positive. This is the case when

(15a) $$\begin{array}{*{20}l} \tau & >-4\pi\lambda_{p}R_{a}\,, \end{array} $$

(15b) $$\begin{array}{*{20}l} J+4\pi\lambda_{p}\left(R_{a}-R_{p}\right) & >0\,. \end{array} $$

The condition *τ*>−4*πλ*_*p*_*R*_*a*_ thus determines an important parameter range in which the circular shape is stable. When the circular shape is unstable, the shape of the cell is hard to predict and will critically depend on the specific implementation of the cell surface and surface dynamics within the diverse CSM model formalisms. Even though the cell shape will be unpredictable, in this parameter regime there is no inherent conflict between fulfilling the area constraint (equivalent to solving *Π*(*a*^∗^)=0) and fulfilling the effective perimeter constraint (equivalent to solving *γ*(*p*^∗^)=0); hence, it is expected that for the shape that will be taken up, *a*^∗^=*A* and *p*^∗^=*P*−*J*/(2*λ*_*p*_). In contrast, when the circular shape is stable, we can analyse the cell radius equilibria for any CSM implementation, as follows next.

#### Cell equilibrium radii and their stability

**Figure 2:**
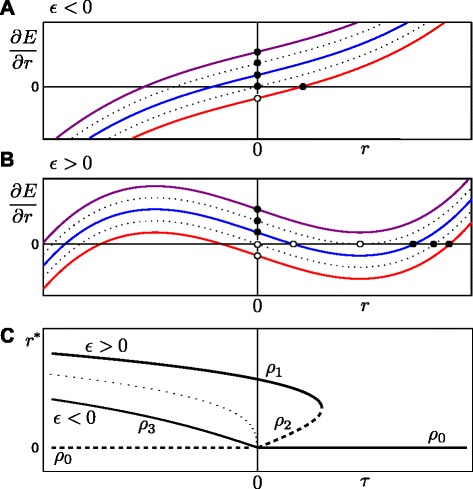
Derivation of possible equilibrium cell sizes using the shape of the energy gradient function *∂* *E*/*∂* *r*.**(A)** The energy gradient function *∂* *E*/*∂* *r* for *ε*<0. The lower and upper dotted lines refer to *τ*=0 and $\tau =4\pi \lambda _{a}\epsilon ^{\frac {3}{2}}$, respectively. For *τ*<0 there is one stable positive equilibrium (red line), otherwise *r* ^∗^=0 is the only stable equilibrium (blue and purple lines). Stable equilibria are indicated by solid circles, unstable equilibria by open circles. **(B)** The energy gradient function *∂* *E*/*∂* *r* for *ε*>0. The lower and upper dotted lines refer again to *τ*=0 and $\tau =4\pi \lambda _{a}\epsilon ^{\frac {3}{2}}$, respectively. For *τ*<0, only one stable equilibrium exists (red line). The interval $0<\tau <4\pi \lambda _{a}\epsilon ^{\frac {3}{2}}$ defines the bistable region (blue line); when $\tau >4\pi \lambda _{a}\epsilon ^{\frac {3}{2}}$, *r* ^∗^=0 is the only stable equilibrium (purple line). The lines in **(A)** and **(B)** have the same parametrisation, except for *ε*, which has the opposite sign. **(C)** Bifurcation diagram showing the cell equilibria as a function of *τ*. Stable equilibria are indicated by solid lines, unstable equilibria by dashed lines. The solutions *ρ*_1_, *ρ*_2_ and *ρ*_3_ are defined by Eq. 19, Eq. 20 and Eq. 21; whereas the trivial solution is defined by *ρ*_0_=0.

The cell equilibria radii are determined by finding the radii for which *∂E*/*∂r*=0, which involves the solving of a reduced cubic equation on *r*. We start by rewriting Eq. 8, separating the terms that are dependent on *r* from the *r*-independent term, *τ* (see Figure 2):

(16) $$  \begin{array}{ccccccc} \frac{\partial E}{\partial r} & = & 2\pi&\left(\underbrace{J-4\pi\lambda_{p}R_{p}}\right. & + & \left.\underbrace{4\pi\lambda_{p}r+2\pi\lambda_{a}r\left(r^{2}-{R_{a}^{2}}\right)} \right)\,,\\ \frac{\partial E}{\partial r} & = & 2\pi&\left(\tau {\vphantom{\frac{0}{0}}}\right.& + & \left. 2\pi\lambda_{a}r\left(r^{2}-3\epsilon\right) \right)\,. \end{array}  $$

With Eq. 16 we can assess how the number of equilibria depends on the two composite parameters, *τ* and $\epsilon =\frac {{R_{a}^{2}}}{3}-\frac {2\lambda _{p}}{3\lambda _{a}}$. If *ε*<0, the right-hand side term 2*πλ*_*a*_*r*(*r*^2^−3*ε*) cannot become negative (it is nonsensical to consider *r*<0), so by itself this term does not give rise to non-trivial equilibria. The only non-trivial equilibrium then occurs when *τ* balances the monotonically increasing right-hand side term (see Figure 2A). This solution is unique and stable, the equilibrium radius is positive if *τ*<0 (*r*^∗^=*ρ*_3_, Figure 2C), but negative and therefore nonsensical if *τ*>0. Consequently, when *ε*<0 and *τ*>0 the only equilibrium is the trivial solution *r*^∗^=*ρ*_0_=0, which is stable, while when *τ*<0 the trivial solution *r*^∗^=*ρ*_0_=0 is unstable (Figure 2A, C). In contrast, if *ε*>0, the right-hand side term 2*πλ*_*a*_*r*(*r*^2^−3*ε*) is not monotonically increasing and hence there can be three real roots (see Figure 2B). Nevertheless, the possible equilibria, their sign and their stability can be easily understood if one recognizes that modifying the value *τ* corresponds to a vertical shift in the function *∂E*/*∂r* (Figure 2A, B). By starting with *τ*=0 (for which *∂E*/*∂r* is an antisymmetric function) and then shifting the graph vertically, all possible equilibria combinations occur successively (Figure 2A, B). For two parameter combinations the number of equilibria changes (Figure 2B, not taking negative *r*-values into account). The lower dotted line shows the transition, at *τ*=0, from two to three equilibria, while the upper dotted line shows the transition from three equilibria to one equilibrium. The latter transition corresponds to a fold bifurcation, while the former transition is a non-standard bifurcation that bears resemblance to a transcritical bifurcation, but formally does not correspond to one. We will therefore henceforth refer to it as a ‘pseudo-transcritical’ bifurcation. The fold bifurcation takes place when the minimum of the *∂E*/*∂r* function equals zero. The radius at which *∂E*/*∂r* has a minimum is given by $r=\sqrt {\epsilon }$, which can be easily determined by setting $\frac {\partial ^{2}E}{\partial r^{2}}=0$:

(17a) $$\begin{array}{*{20}l} \frac{\partial^{2}E}{\partial r^{2}} &=2\pi\left(2\pi\lambda_{a}\left(r^{2}-3\epsilon\right)+4\pi\lambda_{a}r^{2}\right)\,, \end{array} $$

(17b) $$\begin{array}{*{20}l} &=2\pi\left(2\pi\lambda_{a}\left(3r^{2}-3\epsilon\right)\right)\,, \end{array} $$

(17c) $$\begin{array}{*{20}l} &=12\pi^{2}\lambda_{a}\left(r^{2}-\epsilon\right)\,. \end{array} $$

Therefore, the transition from two to zero positive equilibria takes place when

(18) $$\begin{array}{@{}rcl@{}} \left.\frac{\partial E}{\partial r}\right|_{r=\sqrt{\epsilon}}=0 & \Leftrightarrow & \tau=4\pi\lambda_{a}\epsilon^{\frac{3}{2}}\,, \end{array} $$

when *ε*>0. (As discussed above, when *ε*<0, $\frac {\partial E}{\partial r}$ is monotonically increasing, which means that there is no such transition, see Figure 2A).

The stability of the solutions is given by the second derivative (Eq. 17). When *τ*<0, the non-trivial solution *ρ*_1_ is stable while the trivial solution, *r*^∗^=*ρ*_0_=0, is unstable; when *τ*>0, both the non-trivial solution *ρ*_1_ and the trivial solution, *r*^∗^=*ρ*_0_=0 are stable, while the non-trivial solution *r*^∗^=*ρ*_2_ is unstable (Figure 2C).

To obtain convenient expressions of the above-mentioned equilibria, we define here the aggregate parameter $\alpha =\left.\frac {\partial E}{\partial r}\right |_{r=\sqrt {\epsilon },\tau =0}=-8\pi ^{2}\lambda _{a}\epsilon ^{\frac {3}{2}}$. When *ε*>0, $\tau <-\frac {\alpha }{2\pi }$, the stable non-trivial positive equilibrium is given by the following equation (note that in this case *α* is a negative real number)

(19a) $$\begin{array}{@{}rcl@{}} \rho_{1} & =&2\sqrt{\epsilon}\cos\left(\frac{1}{3}\arccos\left(\frac{2\pi\tau}{\alpha}\right)\right)\,. \end{array} $$

When $\tau <\frac {\alpha }{2\pi }$ the above equation is still correct, but inconvenient if used in a non-symbolic computational environment. This is because in this parameter regime $\arccos \left (\frac {2\pi \tau }{\alpha }\right)$ returns a complex number, with the cos() operation returning again real numbers. Therefore, in order to prevent calculations involving complex numbers in the intermediate step, we rewrite *ρ*_1_ for $\tau <\frac {\alpha }{2\pi }$ as (again, note that *α* is still a negative real number)

(19b) $$\begin{array}{@{}rcl@{}} \rho_{1,alt} & =2\sqrt{\epsilon}\cosh\left(\frac{1}{3}\text{arccosh}\left(\frac{2\pi\tau}{\alpha}\right)\right)\,. \end{array} $$

Note that both equations are mathematically equivalent, differing only in how promptly they can be numerically evaluated. When *ε*>0, $0<\tau <-\frac {\alpha }{2\pi }$, the second non-trivial equilibrium, which is non-stable (again, *α* is a negative real number), is given by

(20) $$\begin{array}{@{}rcl@{}} \rho_{2} & =2\sqrt{\epsilon}\sin\left(\frac{1}{3}\arcsin\left(-\frac{2\pi\tau}{\alpha}\right)\right)\,. \end{array} $$

Finally, as discussed above, when *ε*<0, *τ*<0 there is only one non-trivial positive equilibrium, which is stable (Figure 2A). In this case *α* is a positive imaginary number. We therefore introduce another aggregate parameter *ζ*, which is positive and real when *ε*<0, namely $\zeta =-\left (\frac {\alpha }{2\pi }\right)^{2}$. The equilibrium radius is then given by

(21) $$  \rho_{3} =\frac{\sqrt{-\epsilon}}{\sqrt[6]{\zeta}}\left(\sqrt[3]{-\tau+\sqrt{\tau^{2}+\zeta}}-\sqrt[3]{\tau+\sqrt{\tau^{2}+\zeta}}\right)\,.  $$

**Figure 3:**
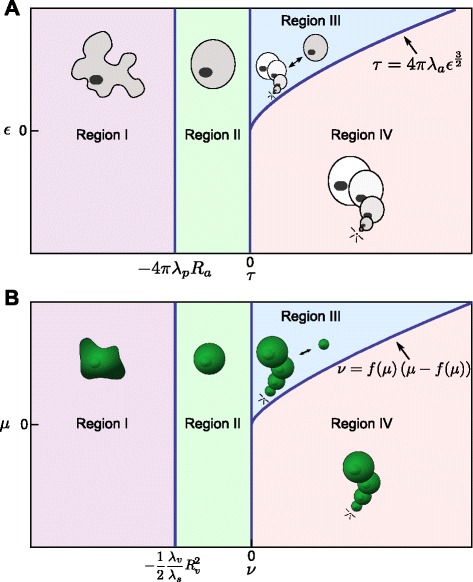
All possible dynamics for a 2D circular cell and a 3D spherical cell within the CSM formalisms can be captured by two aggregate biophysical parameters.**(A)** Bifurcation diagram showing the dynamics as a function of *τ* and *ε*. Region I (purple area): the interfacial tension is negative. The absence of positive tension along the membrane will lead to an unpredictable cell shape, at which *a* ^∗^=*A*, and *p* ^∗^=*P*−*J*/(2*λ*_*p*_). Region II (green area): The cell has a positive interfacial tension at the equilibrium radius. However, when the radius is sufficiently smaller than the equilibrium radius, the interfacial tension is negative. Region III (blue area): a circular cell shape is always stable. There are two possible stable equilibria (bistable regime), a positive *r* ^∗^=*ρ*_1_ and *r* ^∗^=0, separated by an energy maximum at *ρ*_2_. Region IV (pink area): the only stable equilibrium is *r* ^∗^=0, i.e. cells shrink and disappear. **(B)** Bifurcation diagram for a 3D spherical cell, plotted as a function of the aggregate parameters *μ* and *ν*. Qualitatively, the same four regions as obtained for the 2D case are observed for the spherical cell. Again, the cell behaviour is fully determined by two degrees of freedom, here shown for the (*ν*,*μ*)-space.

The parameter conditions for the four possible dynamic behaviours of a 2D circular shaped cell are summarised in Table 1. These conditions determine regions of different cell behaviour, as shown in Figure 3A. The analysis reveals that, within a cell surface mechanics description, all possible cell behaviours are captured by four modes only, and that these can be distinguished based only on the two aggregate parameters, *τ* and *ε*.

**Table 1:** **Parameter conditions for the four possible dynamic behaviours of a 2D circular shaped cell**

**Region**	**Condition(s)**
I	*τ*<−4*π* *λ*_*p*_ *R*_*a*_
II	−4*π* *λ*_*p*_ *R*_*a*_<*τ*<0
III	$0<\tau <4\pi \lambda _{a}\epsilon ^{\frac {3}{2}}$
IV	$\tau >4\pi \lambda _{a}\epsilon ^{\frac {3}{2}}$

### 3D spherical cell

When an equivalent analysis is performed for the 3D case, we again find that there are only four qualitatively different behaviours possible for a single cell. Moreover, these behaviours can again be fully determined by only two aggregate parameters. Below, we briefly present the outline of the analysis, leaving the details for Appendix A.

#### Energy function, interfacial tension and pressure

In 3D, the energy function becomes

(22) $$ E\left(s,v\right|J,\lambda_{s},S,\lambda_{v},V)=Js+\lambda_{s}\left(s-S\right)^{2}+\lambda_{v}\left(v-V\right)^{2}\,,  $$

where *s* and *v* are the surface area and volume of the cell, respectively. Again, five parameters describe the cellular properties: *J*, an energy per surface area unit due to adhesion with the surrounding medium or neighbouring cells; *S*, the membrane rest surface area; *V*, the target cell volume. The constraints are modulated by the Lagrange multipliers *λ*_*s*_ and *λ*_*v*_, which weigh the relative tension contributions of actin-myosin contraction and cell deformations, respectively. Again we consider that *J*-values can be both positive and negative, but that negative values for the Lagrange multipliers are nonsensical. Using the surface and volume of a sphere (*s*=4*πr*^2^ and $v=\frac {4}{3}\pi r^{3}$ respectively), we re-write *E* for a spherical cell as a function of *r*,

(23) $$  E=4\pi Jr^{2}+\lambda_{s}\left(4\pi r^{2}-4\pi {R_{s}^{2}}\right)^{2}+\lambda_{v}\left(\frac{4}{3}\pi r^{3}-\frac{4}{3}\pi {R_{v}^{3}}\right)^{2}\,,  $$

where $R_{s}=\frac {1}{2}\sqrt {\frac {S}{\pi }}$ and $R_{v}=\sqrt [3]{\frac {3V}{4\pi }}$. As before, interfacial tension and pressure can be defined as

(24a) $$\begin{array}{*{20}l} \gamma &= \ \ \frac{\partial E}{\partial s} \ \hspace*{1pt} = J+8{\pi}\lambda_{s}\left(r^{2}-{R_{s}^{2}}\right)\,, \end{array} $$

(24b) $$\begin{array}{*{20}l} \Pi &= -\frac{\partial E}{\partial v} = -\frac{8}{3}\pi\lambda_{v}\left(r^{3}-{R_{v}^{3}}\right)\,. \end{array} $$

Again, the interfacial tension has a surface-area-dependent and a surface-area-independent component, 8*πλ*_*s*_*r*^2^ and $J-8\pi \lambda _{s}{R_{s}^{2}}$, respectively. As in 2D, we denote the surface-area-independent component of the interfacial tension by *τ*, i.e. $\tau =J-8\pi \lambda _{s}{R_{s}^{2}}$ and *γ*=*τ*+8*πλ*_*s*_*r*^2^. (Note that the expression for *τ* differs from the 2D case.)

#### Cell equilibrium radii, their interfacial tension and stability

The first step is to determine whether the interfacial tension at an equilibrium radius is expected to be positive. As discussed for the 2D case, this does not require specific knowledge regarding the radius at equilibrium. Following a comparable analysis as performed for the 2D case, we derive in Appendix A the following condition for a positive interfacial tension in 3D:

(25) $$ \tau>-8\pi\lambda_{s}{R_{v}^{2}}\,,  $$

which is very comparable to the condition expressed in Eq. 14b for the 2D case.

In the next step we look at the equilibrium radii themselves. As for the 2D case, the cell radii are determined by finding the radii for which *∂E*/*∂r*=0, which now involves solving a quintic equation of *r*. In Appendix A we show that the derivative of *E* with respect to the radius of the cell can be written as

(26) $$ \frac{\partial E}{\partial r}=8\pi\left(ar^{5}+br^{3}-cr^{2}+\tau r\right)\,,  $$

where *τ* is, as previously, the surface-area-independent part of the interfacial tension, here given by $J-8\pi \lambda _{s}{R_{s}^{2}}$, which can be either positive or negative; and the other lumped parameters are respectively $a=\frac {4}{3}\pi \lambda _{v}$, *b*=8*πλ*_*s*_, and $c=\frac {4}{3}\pi \lambda _{v}{R_{v}^{3}}$, with *a* strictly positive and *b* and *c* strictly non-negative for any reasonable parameter choice.

The first observation is that $\left.\frac {\partial E}{\partial r}\right |_{r=0}=0$ for any parameter choice. This is an important difference with the 2D case. It implies that in 3D, a cell with a radius close to zero slows down its rate of shrinkage or expansion (depending on the stability of the zero-radius equilibrium), while in 2D, the rate of shrinkage or expansion typically remains non-zero for very small radii (given by $\left.\frac {\partial E}{\partial r}\right |_{r=0}=\tau $). This difference has a clear impact on the cell dynamics, and plays a role in computational implementations of CSM formalisms.

The closed-form solutions to $\frac {\partial E}{\partial r}=0$ for the 3D case are not included here because their full expressions are hardly informative. Note, however, that its numerical evaluation can be easily performed if needed.

As in 2D, the number of equilibria and their stability depend on the sign of *τ*. If *τ* is negative, there is only one stable positive equilibrium radius, whereas if *τ* is positive two scenarios are possible: no equilibrium at all or two non-trivial equilibria, one lower and unstable, and another greater and stable, as summarized in Figure 3B, and derived in Appendix A.

The two regimes for which *τ*>0 are separated by a fold bifurcation. The transition from two to no equilibria implies that, at the bifurcation, $\left.\frac {\partial E}{\partial r}\right |_{r=r^{*}}=0$ and $\left.\frac {\partial ^{2}E}{\partial r^{2}}\right |_{r=r^{*}}=0$. The bifurcation lines that separate all the possible dynamical regimes can be captured by defining a two-dimensional parameter (*μ*,*ν*)-plane (see Appendix A), where $\nu =\frac {\left (J-8\pi \lambda _{s}{R_{s}^{2}}\right)\lambda _{v}}{16{\pi \lambda _{s}^{2}}}=\frac {\tau \lambda _{v}}{16{\pi \lambda _{s}^{2}}}=\frac {3a\tau }{b^{2}}$, and $\mu =\frac {{R_{v}^{3}}}{8\left (\frac {\lambda _{s}}{\lambda _{v}}\right)^{\frac {3}{2}}}$. In this plane, the bifurcation lines are defined by

(27a) $$\begin{array}{*{20}l} \nu & =-\frac{\lambda_{v}{R_{v}^{2}}}{2\lambda_{s}}\,, \end{array} $$

(27b) $$\begin{array}{*{20}l} \nu & =0\,, \end{array} $$

(27c) $$\begin{array}{*{20}l} \nu & =f(\mu)\left(\mu-f(\mu)\right)\,, \end{array} $$

where $f(\mu)=\sinh \left (\frac {1}{3}\operatorname {arcsinh}\left (\mu \right)\right)$. Figure 3B shows the full 3D bifurcation diagram.

Thus, by calculating *μ* and *ν*, which are dependent on the parameters of the CSM, one can immediately establish (without the need for simulations) the expected behaviour of a single spherical cell. The distinguishing feature from the 2D case regards the dynamics (but not the stability) close to zero radius.

### Cellular Potts model (CPM)

The CPM, a prominent member of the class of cell surface mechanics formalisms, distinguishes itself by being a lattice-based formalism. This property requires special attention on how to relate the CPM numerical descriptors to the variables of the analytical description of CSM. Specifically, it is crucial to explain how the variables *p* and *a* of the analytical description relate to the lattice-based descriptors of perimeter and area in a CPM simulation.

A cell within the CPM consists of a set of lattice (or grid) points that together define a region in a two dimensional (2D) or three dimensional (3D) lattice. In 2D, the set of lattice points (*i*,*j*) that define a specific cell are indicated by a unique identifier *σ*_*i*,*j*_. There are as many possible cell shapes as the degrees of freedom defined by the number of permutations of lattice points composing the cell *σ*, i.e. the bulk area $n_{\sigma }=\sum _{\textit {ij}}\delta _{\sigma _{\textit {ij}},\sigma }$ (where *δ* is Kronecker’s delta function). This is an important feature of the CPM compared to other methods that treat cells as points or centre-of-mass based entities, as it allows an indefinite freedom for cell deformation, being only limited by the spatial resolution. Although many CPM studies explicitly relate the lattice spatial unit *Δx* to a physical length (e.g. 1 *μm*), relevant model parameters, such as target area or adhesion energies are still often expressed in terms of the lattice arbitrary spatial unit only. To link such lattice-point-based parameters to the above analysis, as well as to understand how a change in spatial resolution affects the parameters, we here explicitly take the resolution of the lattice *k*=1/*Δx* into account. We thus distinguish between the bulk area *n*_*σ*_, expressed in number of lattice points, and the actual area of a CPM cell *a*_*σ*_, expressed in *Δx*^2^. These two areas only differ in that *n*_*σ*_ is the number of lattice points composing the CPM cell *σ*, whereas *a*_*σ*_=*n*_*σ*_/*k*^2^ is the physical area of a CPM cell, and *k* the factor describing the resolution of the lattice. The area *a* in the analytical description maps to the CPM area *a*_*σ*_. The explanation on how the CPM cell perimeter *p*_*σ*_ relates to the perimeter *p* of the analytical description is not as simple as for the area, and is therefore deferred to the next section.

In a CPM simulation the cell shape dynamics are calculated using a Monte Carlo algorithm. In a random sampling through the lattice, for each lattice point a local shape change $(\sigma _{\textit {ij}}\rightarrow \sigma _{i^{\prime }j^{\prime }})\phantom {\dot {i}\!}$ is considered, i.e. a protrusion/retraction of a cell into a neighbouring cell or medium. An energy change $\Delta E = E(\sigma _{i^{\prime }j^{\prime }})- E(\sigma _{\textit {ij}})$ is calculated which defines a probability $p(\sigma _{\textit {ij}}\rightarrow \sigma _{i^{\prime }j^{\prime }})$ for that event to happen,

(28) $$ p(\sigma_{ij}\rightarrow\sigma_{i^{\prime}j^{\prime}})=\left\{ \begin{array}{ccc} 1 & \text{if} & \Delta E<-Y\,,\\ e^{\left(-\frac{\Delta E+Y}{T}\right)} & \text{if} & \Delta E\geq-Y\,. \end{array}\right.  $$

The energy function *E* from which the dynamics are computed is either Eq. 1 for 2D lattices or Eq. 22 for 3D lattices, as explained previously. In Eq. 28, the parameter *Y* represents a yield – the ability of the membrane to resist a force, in many studies, including this one, set to zero – and *T* a simulation temperature which determines the extent of fluctuations. The higher the temperature, the more probable it is to observe energetically unfavourable cell shape deformations.

#### Contact length perimeter and contact surface area in the CPM

**Figure 4:**
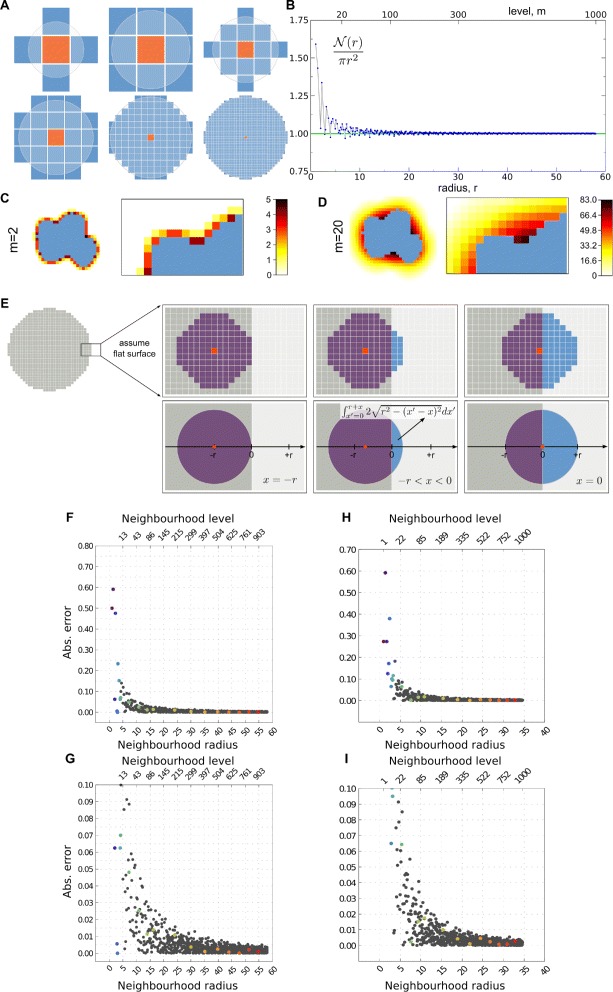
Perimeter determination in a lattice-based environment like CPM.**(A)** Examples of neighbourhoods. From left to right, top to bottom: von Neumann neighbourhood (*m*=1), Moore neighbourhood (*m*=2), third-, fourth-, twentieth- and hundredth-level neighbourhoods. Orange site indicates origin, neighbourhood (sites in blue) is determined by the neighbourhood radius (light blue circle). **(B)** Lattice points within a certain radius, relative to the expected area of a circle, as a function of neighbourhood radius. *m* indexes the corresponding neighbourhood levels. **(C**, **D)** Cell neighbouring regions used for perimeter-related computations in the CPM, for neighbourhood levels *m*=2**(C)** and *m*=20**(D)**. Colour gradients denote the number of times neighbouring lattice points are taken into account in the perimeter computation. **(E)** The CPM cell perimeter in 2D (or surface area in 3D) is calculated by summing all lattice points falling within the given neighbourhood which have a different state (blue lattice points) than that of the cell (grey lattice points), for all lattice sites of the cell (red lattice points). The upper row illustrates the discrete process as is performed in a CPM simulation, while the lower row illustrates the continuous approximation of this process. **(F**–**I)** Precision of the CPM perimeter correction factor *ξ* depends on the neighbourhood level used in the CPM simulations. The graphs show the fractional difference between the numerical and analytical estimate of *ξ* as a function of the neighbourhood radius. **(F**, **G)** 2D numerical estimate compared with Eq. 29. **(H**, **I)** 3D numerical estimate compared with Eq. 33. For the numerical estimates a flat cell boundary is used with increasing neighbourhood levels, from 1 to 1,000. **(G)** and **(I)** are magnifications of **(F)** and **(H)**, respectively.

The CPM cell perimeter *p*_*σ*_ does not have a straightforward connection to the perimeter *p* (or surface area *s* in 3D) of the analytical description. In fact, the CPM cell perimeter is a source of much confusion and implementation errors within the community, requiring clarification. The source of the confusion stems from the fact that there is no direct way of mapping the perimeter or surface area of a cell onto a 2D or 3D lattice (although the area (in 2D) and volume (in 3D) are readily available). When determining the (local) perimeter, one can not simply take the total edge length between neighbouring lattice points belonging to different cells, because this leads to large grid effects, with diagonally oriented edges presenting a $\sqrt {2}\approx 41\%$ larger edge length than horizontally or vertically oriented edges [35,42]. It could seem that the cell’s discrete boundary could be approximated by a continuous description, i.e. using interpolation techniques. However, not only can these techniques be computationally expensive, they also presume that there is a clear definition of the ordered set of points belonging to the cell boundary, which, for two reasons, does not hold for the CPM. Firstly, the CPM does not keep track of “boundary sites”, the boundary being solely a consequence of the set of lattice points constituting the cell, therefore not providing any ordering. Secondly, the boundary of a CPM cell is not necessarily a single simple closed curve. Rather, the fraction of lattice points occupied by the cell drops from unity to zero when passing the edge of the cell, allowing for discontinuities in the boundary at the level of the lattice site, while still capturing a single, continuous boundary at the interface level. Thus, interpolation techniques render themselves ill-defined in the context of the CPM, and can therefore not be used. In short, the perimeter *p* (or surface area *s* in 3D) does not follow directly from the lattice where cells are embedded. Nevertheless, it is an important quantity which has to be determined in CPM simulations, because both the adhesion-driven tension and the cortical tension depend on it. Therefore an alternative method has to be used to compute the perimeter in the CPM, to ensure that comparisons can be drawn between the CPM and the more general CSM. To understand how to correctly calculate it, we have to look at how these tensions are implemented in the CPM formalism. Solving the forces is done implicitly via the energy variation *ΔE* caused by a local deformation $(\sigma _{\textit {ij}}\rightarrow \sigma _{i^{\prime }j^{\prime }})\phantom {\dot {i}\!}$, which involves an effective computation of the local perimeter variations, i.e. $\Delta p=p_{\sigma _{i^{\prime }j^{\prime }}}-p_{\sigma _{\textit {ij}}}\phantom {\dot {i}\!}$. Instead of calculating *Δp* in terms of a change of contour length, a local neighbourhood is defined around the lattice point *σ*_*i*,*j*_ for which the state change is evaluated. Then the algorithm sweeps all the lattice points defined by this neighbourhood, summing the number of lattice points that do not belong to this cell before and after the evaluated state change to obtain a measure of the change in contact between the cell and its neighbours. Usually, a radial neighbourhood is used. Figure 4A shows neighbourhoods of different sizes. The set of lattice points (*x*,*y*) around a site (*x*_0_,*y*_0_) that form the neighbourhood is determined by the neighbourhood radius: $\mathcal {N}\left (r\right)=\{\left (x,y\right):\sqrt {(x-x_{0})^{2}+(y-y_{0})^{2}}\le r\}$, where *r* is the lowest radius that satisfies this inequality [43]. Note that often neighbourhood level rather than radius is used, based on the sequence of all possible neighbourhoods ranked by size. It is clear that while the neighbourhood is grid-based, at larger neighbourhoods the shape approximates a circle. This is quantified in Figure 4B, indicating that the area divided by *πr*^2^, the expected area if it were a circle, converges to unity for larger neighbourhood levels. In the CPM the perimeter of the cell is approximated by counting for each lattice point belonging to the cell within the predefined neighbourhood all the lattice points not belonging to that cell. Therefore this computation involves two nested loops, each with the algorithmic complexity of the specific spatial dimensions, i.e., $\mathcal {O}(n^{2})$ for 2D and $\mathcal {O}(n^{3})$ for 3D. This approach leads to neighbouring lattice points being counted multiple times. Figure 4C illustrates this effect when a second level (or Moore) neighbourhood is being used. While for this configuration some lattice points are counted only once, others are counted up to 5 times. For a 20th level neighbourhood (Figure 4D), there is an effective zone around the cell, with decreasing contribution to the cell’s perceived perimeter at larger distance from the cell. So how can such a proxy for perimeter be related to the geometric length measure? To make the link between the perceived perimeter and the true perimeter, we consider a cell whose cell edge can be approximated by a non-curved interface at the scale of the neighbourhood, while the neighbourhood itself is sufficiently large to be considered to be more or less circular. Figure 4E illustrates for such a scenario how the perceived perimeter within the CPM is calculated. It shows for a number of lattice points, indicated in red, their contribution to the perceived perimeter, indicated in blue. The upper row illustrates the discrete process as is performed in a CPM simulation, while the lower row illustrates a continuous approximation of this process. Two important features are noteworthy. First, any lattice point whose distance to the cell edge lies within the neighbourhood radius contributes to the perceived perimeter, but the lattice points close to the cell boundary contribute more than the lattice points further away from the boundary. Secondly, each lattice point should be regarded to occupy a specific area. Given that for each lattice point this area is multiplied with the area within its neighbourhood that extends outside of the cell, the used proxy for perimeter length in the CPM, $p_{_{\mathit {CPM}}}$, has dimensions of area times area, or *L*^4^, where *L* indicates dimension of the quantity length. This implies that a correction factor *ξ* (with dimension *L*^3^) is needed to scale back from this proxy with dimension *L*^4^ to the effective length *p*, with dimension *L*. This correction factor corresponds to a scaling factor *ξ* that multiplies per infinitesimal perimeter length. For large neighbourhoods, in 2D, this factor converges to:

(29) $$ \xi=\int_{x{=}-r}^{0}\int_{x'{=}0}^{r{+}x}2\sqrt{r^{2}-(x'-x)^{2}}dx'dx=\frac{2}{3}r^{3}\,,  $$

as illustrated in Figure 4E. In this equation, *r* indicates the radius of the used neighbourhood; *x* gives the position of the ‘lattice sites’ contributing to the perceived perimeter (red dots in Figure 4E), relative to the cell edge, with negative values indicating internal and positive values indicating external to the cell; and *x*^′^ is varied over the interval [0,*r*+*x*], defining the distance to the cell edge within the neighbourhood’s protruding region (i.e., the position within the blue circular segments in Figure 4E). The first integral captures the fact that contributions to the perceived perimeter are generated by lattice sites located from a distance equivalent to the neighbourhood radius inwards, up to the cell edge. The second integral captures the area of the neighbourhood extending outside of the cell. Often in CPM studies, the neighbourhood level instead of the radius is reported. Therefore, in order to determine *ξ* via Eq. 29, the level has to be mapped to the radius first (see Table 2). This problem is equivalent to the sum of two squares representation, and hence an analytic expression for such a conversion does not exist, neither in 2D nor in 3D, and needs to be calculated iteratively [44]. Once *ξ* is determined, the 2D actual geometric perimeter *p* can be approximated by

(30) $$ p=\frac{p_{_{\mathit{CPM}}}}{\xi}\,.  $$

**Table 2:** **Theoretical and measured values of the perimeter scaling factor** ***ξ*** **for small neighbourhood sizes, needed for calculating the effective** ***J*** **value in the CPM**

	**2D**				**3D**			
**neigh.**	**neigh.**	***ξ*** **, estimated**	***ξ*** **, numer.**	**% error**	**neigh.**	***ξ*** **, estimated**	***ξ*** **, numer.**	**% error**
**number**	**radius**	**using Eq.** 29	**determined**		**radius**	**using Eq.** 33	**determined**	
1	1	0.67	1	50.00	1	0.79	1	27.32
2	$\sqrt {2}$	1.89	3	59.10	$\sqrt {2}$	3.14	5	59.15
3	2	5.33	5	-6.25	$\sqrt {3}$	7.07	9	27.32
4	$\sqrt {5}$	7.45	11	47.58	2	12.57	11	-12.46
5	$2\sqrt {2}$	15.08	15	-0.56	$\sqrt {5}$	19.64	23	17.14
6	3	18.00	18	0	$\sqrt {6}$	28.27	39	37.93
7	$\sqrt {10}$	21.08	26	23.33	$2\sqrt {2}$	50.27	47	-6.50
8	$\sqrt {13}$	31.25	36	15.21	3	63.62	70	10.03
12	$2\sqrt {5}$	59.63	68	14.04	$\sqrt {13}$	132.73	134	0.96
20	$2\sqrt {10}$	168.66	173	2.58	$\sqrt {22}$	380.13	410	7.86
100	$3\sqrt {29}$	2811.06	2850	1.39	$\sqrt {118}$	1.09·10^4^	11127	1.75
1000	$\sqrt {3365}$	130133	130181	0.037	$3\sqrt {133}$	1.13·10^6^	1127539	0.20

Given that *Jp* equates to $J_{_{\mathit {CPM}}}p_{_{\mathit {CPM}}}$, it follows that for the actual adhesion energy

(31a) $$\begin{array}{@{}rcl@{}} J=\xi J_{_{\mathit{CPM}}}\,. \end{array} $$

Similar relationships for the actual geometric membrane rest length and perimeter constraint follow, namely

(31b) $$\begin{array}{@{}rcl@{}} P =\frac{P_{_{\mathit{CPM}}}}{\xi}\,, \end{array} $$

(31c) $$\begin{array}{@{}rcl@{}} \lambda_{p} =\xi^{2}\lambda_{p\,{}_{\mathit{CPM}}}\,. \end{array} $$

Reciprocally, known adhesion energies, membrane rest lengths, and perimeter constraints, respectively, can be appropriately scaled within the CPM formalism by applying the corrections

(32a) $$\begin{array}{*{20}l} J_{_{\mathit{CPM}}} & =\frac{J}{\xi}\,, \end{array} $$

(32b) $$\begin{array}{*{20}l} P_{_{\mathit{CPM}}} & =\xi P\,, \end{array} $$

(32c) $$\begin{array}{*{20}l} \lambda_{p\,{}_{\mathit{CPM}}} & =\frac{\lambda_{p}}{\xi^{2}}\,. \end{array} $$

In 3D, the proxy used for surface area in CPM simulations has dimensions volume times volume, or *L*^6^, while the required correction factor *ξ* to scale back to the effective surface area, with dimensions *L*^2^ is given by

(33) $$  \xi=\int_{x{=}-r}^{0}\int_{x'{=}0}^{r{+}x}\pi\left(r^{2}-(x'-x)^{2}\right)dx'dx=\frac{\pi}{4}r^{4}\,.  $$

The ways to scale *s*, *J*, *S*, and *λ*_*s*_ between the geometric and CPM parameters in 3D are analogous to the scaling of *p*, *P*, and *λ*_*p*_ in 2D,

(34a) $$\begin{array}{*{20}l} s &=\ \ \ \ \frac{s_{_{\mathit{CPM}}}}{\xi}\,, \quad \ \ \ \ s_{_{\mathit{CPM}}} =\xi s\,, \end{array} $$

(34b) $$\begin{array}{*{20}l} J &=\ \ \ \ \xi J_{_{\mathit{CPM}}}\,, \quad \ \ \hspace*{3pt} J_{_{\mathit{CPM}}} =\frac{J}{\xi}\,, \end{array} $$

(34c) $$\begin{array}{*{20}l} S &=\ \ \ \ \frac{S_{_{\mathit{CPM}}}}{\xi}\,, \quad \ \ \hspace*{2pt} S_{_{\mathit{CPM}}} =\xi S\,, \end{array} $$

(34d) $$\begin{array}{*{20}l} \lambda_{s} &=\xi^{2}\lambda_{s\,{}_{\mathit{CPM}}}\,,\quad \lambda_{s\,{}_{\mathit{CPM}}} =\frac{\lambda_{s}}{\xi^{2}}\,. \end{array} $$

If one aims for a fair comparison between CPM simulations and either the analytical results shown throughout this paper, or the simulations performed with other CSM-based formalisms, the factor *ξ* has to be determined for the chosen neighbourhood and the parameters adapted accordingly. Moreover, without applying such a correction no direct quantitative link can be made between biophysical parameters used in CPM simulations and experimental measurements.

The analytical results given in Eq. 29 and Eq. 33 assume a perfectly circular neighbourhood. Small neighbourhoods can deviate substantially from this approximation, as shown in Figure 4B. We therefore measured the residual error in perimeter length after applying the correction factor. Figure 4F and H show the residual error for 2D and 3D, respectively, while Figure 4G and I show the same data magnified. For neighbourhoods with a radius equal or larger than 5, both in 2D and in 3D, the error remains within 6%. For small neighbourhoods, however, the errors can be large, with a 59% mismatch for a 2nd level neighbourhood (see Table 2). Good choices are the 5th level neighbourhood (0.56% error) and the 6th level neighbourhood (0% error). In 3D, the often used 3rd level neighbourhood presents a 27% error, while the 7th level neighbourhood presents a 6% error. In general it is best to use a numerically established correction factor *ξ* for small neighbourhoods, rather than directly applying Eq. 29 and Eq. 33 (see Table 2).

The choice of the neighbourhood is therefore very important, because it defines both the “localness” of the interfacial tension computation as well as its dependence on the grid geometry, which can be a source of angular bias in the cell’s shape [35]. In general, a radial neighbourhood spanning a wider region will take more lattice points into account and will be closer to the circle (or sphere in 3D), leading to a better approximation of the perimeter. However, the neighbourhood should be sufficiently small compared to the size of the cells themselves, as with increasing neighbourhood radius the information integration becomes less local. Hence, there is a trade-off between being local, which is desired as it improves spatial resolution, and isotropy of the computation itself. Moreover, larger neighbourhoods are computationally more costly.

In addition, the same neighbourhood level/radius needs to be used in all computations involving perimeter, such as the energy change calculations stemming from adhesion as well as from perimeter conservation.

In order to prevent anisotropic bias stemming from the neighbourhood choice, one may opt to increase its radius while concurrently increasing the spatial resolution (e.g. a 20th level neighbourhood would already be very close to being perfectly isotropic for most CPM studies). This way, a good compromise between the localness and isotropy of the interfacial tension computation can be achieved.

#### CPM spatial resolution

In many of the previous CPM studies (e.g. [21,45]), the absolute space scale was not always deemed crucial and hence often not reported. However, due to a wider application of the CPM, including its role as a predictive tool of experimental data, it has become important to explicitly state the spatial scale (e.g. [14,46,47]). Particularly, it is often relevant to run simulations at different levels of resolution, to allow for more precision, to reduce anisotropy, or to achieve a different spatial scale of temperature-driven fluctuations (the latter being directly linked to the lattice space scale). We therefore present here how one can easily adapt the simulation parameters to a change in the spatial resolution. After deciding on a certain scaling for each lattice point in the simulation, all parameters should be changed in such a way that the cell dynamics remain similar. This corresponds to deriving a new parametrisation, *J*^′^,*λp*′,*P*^′^,*λa*′,*A*^′^ (or *J*^′^,*λs*′,*S*^′^,*λv*′,*V*^′^ in 3D), such that the energy derivative (Eq. 5) remains equal to that prior to the scaling. When the space scale is refined by a factor *k*, then any 2D cell *σ* with a perimeter *p*_*σ*_ and area *a*_*σ*_ will have both a new perimeter *pσ*′=*kp*_*σ*_ and a new area *aσ*′=*k*^2^*a*_*σ*_ after rescaling by *k*. To keep the balance between the different forces as given in Eq. 5 when the spatial resolution is changed, the following equalities should hold:

(35a) $$\begin{array}{*{20}l} &\Pi\nabla(a)-\gamma\nabla(p) =\Pi\nabla(a')-\gamma\nabla(p')\,, \end{array} $$

(35b) $$\begin{array}{*{20}l} &-\frac{\partial E(p,a)}{\partial a}\nabla(a)-\frac{\partial E(p,a)}{\partial p}\nabla(p)=\\&-\frac{\partial E(p',a')}{\partial a'}\nabla(a') -\frac{\partial E(p',a')}{\partial p'}\nabla(p')\,. \end{array} $$

By substituting $p_{\sigma }^{'}={kp}_{\sigma }$ and $a_{\sigma }^{'}=k^{2}a_{\sigma }$ into Eq. 1, it follows that

(36a) $$\begin{array}{*{20}l} \frac{\partial E(p',a')}{\partial p'} &=J'+2k\lambda'_{p}\left(p-\frac{P'}{k}\right)\,, \end{array} $$

(36b) $$\begin{array}{*{20}l} -\frac{\partial E(p',a')}{\partial a'} &= -2k^{2}\lambda'_{a}\left(a-\frac{A'}{k^{2}}\right)\,, \end{array} $$

which, substituted into Eq. 35b gives

(37a) $$\begin{array}{*{20}l} &-2\lambda_{a}\left(a-A\right)\nabla(a)-\left(J+2\lambda_{p}\left(p-P\right)\right)\nabla(p)\\ &\quad=-2k^{2}\lambda'_{a}\left(a-\frac{A'}{k^{2}}\right)\nabla(k^{2}a)\\ &\quad\quad-\left(J'+2k\lambda'_{p}\left(p-\frac{P'}{k}\right)\right)\nabla(kp)\,, \end{array} $$

(37b) $$\begin{array}{*{20}l} &-2\lambda_{a}\left(a-A\right)\nabla(a)-\left(J+2\lambda_{p}\left(p-P\right)\right)\nabla(p)\\ &\quad=-2k^{4}\lambda'_{a}\left(a-\frac{A'}{k^{2}}\right)\nabla(a)\\ &\quad\quad-\left(kJ'+2k^{2}\lambda'_{p}\left(p-\frac{P'}{k}\right)\right)\nabla(p)\,. \end{array} $$

Hence, in order to generate an equivalence with the cell dynamics before scaling, the following adjustments have to be made to the parameters:

(38a) $$\begin{array}{*{20}l} J' &= J/k\,, \end{array} $$

(38b) $$\begin{array}{*{20}l} \lambda'_{p} &= \lambda_{p}/k^{2}\,, \end{array} $$

(38c) $$\begin{array}{*{20}l} \lambda'_{a} &= \lambda_{a}/k^{4}\,, \end{array} $$

(38d) $$\begin{array}{*{20}l} P' &=kP\,, \end{array} $$

(38e) $$\begin{array}{*{20}l} A' &=k^{2}A\,. \end{array} $$

This analysis can be extrapolated to 3D, giving the following parameter correspondence:

(39a) $$\begin{array}{*{20}l} J' &=J/k^{2}\,, \end{array} $$

(39b) $$\begin{array}{*{20}l} \lambda'_{s} &=\lambda_{s}/k^{4}\,, \end{array} $$

(39c) $$\begin{array}{*{20}l} \lambda'_{v} &=\lambda_{v}/k^{6}\,, \end{array} $$

(39d) $$\begin{array}{*{20}l} S' &=k^{2}S\,, \end{array} $$

(39e) $$\begin{array}{*{20}l} V' &=k^{3}V\,. \end{array} $$

**Figure 5:**
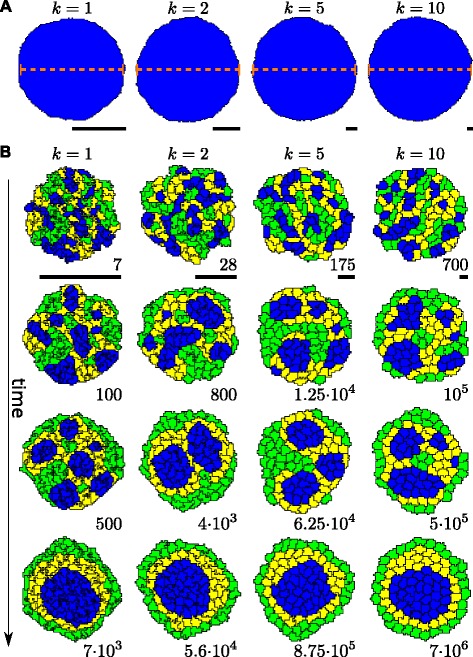
CPM dynamics at different resolutions.**(A)** Simulations of a single cell at different resolutions (*k*=1, 2, 5, 10). For visual comparison, each panel shows the simulation after 10,000 MCS, but rescaled with the *k*-value used for the simulation. See Appendix C for parameter details. Scale bars indicate a length of 50 lattice points. The dashed red lines indicate the analytically predicted cell diameter, a very close correspondence is found for all resolutions. **(B)** Simulations of a tissue containing three different cell types, G(reen), Y(ellow), and B(lue), as well as M(edium), again at four different resolutions. As before, each panel shows the simulation rescaled with the *k*-value used for visual comparison, with time (expressed in Monte Carlo time steps (MCS), in which each point of the lattice is considered for an update once) indicated below, and scale bars representing a length of 50 lattice points. The time required for formation and rounding up of clusters scales with *k* ^2^ (see upper row), while the time required for the drift and merging of small clusters, eventually leading to complete cell sorting, scales with *k* ^3^ (middle rows and lower row). Further parameter details are given in Appendix C.

Note that the validity of this scaling does not depend on any assumptions regarding the shape of cells. Figure 5 shows simulations at different resolutions (*k*∈{1,2,5,10}), with respective scaled parameters. We observe similar cell shape, perimeter length, and area, as well as the same overall patterning, namely the formation of three tissue rings driven by differential-adhesion cell sorting. Note, however, that the time-scale changes, with dynamics slowing down when the spatial resolution increases. For instance, the rounding up of initial clusters, driven by a negative energy gradient and happening on a short time-scale, is, expressed in lattice points, inversely related to the spatial scale, hence *k* times slower in terms of lattice points, but *k*^2^ times slower in terms of actual geometric length (Figure 5B, upper row). The coalescence of small clusters into larger ones happens on an even slower time-scale. This results from the comparatively lower amplitude of the temperature-driven fluctuations when the spatial resolution is increased. Many small, neutral or almost neutral fluctuations allow cells and small clusters to slowly move around, alike a random walk. Therefore, the mean distance travelled by cells, expressed in lattice points, scales with *Δx*^2^=1/*k*^2^, which in actual geometric length implies a slowing down of the displacement by a factor *k*^3^, as shown in the simulations (see Figure 5B, remaining rows). Consequently, simulations at a 10-fold higher resolution evolve 1000 times slower, requiring 10^5^ more computational time in 2D (given that the field is 10×10=100 times larger), and 10^6^ more computational time in 3D. The computational strength of the CPM lies in the fact that an energy change *ΔE* caused by a local shape change can be calculated locally and hence very efficiently, in both both 2D and 3D simulations. As a consequence, both in 2D and 3D computational time scales linearly with the number of cells. The observation that, in sharp contrast, a 10-fold higher resolution requires a 100,000 or even a million times more calculations (in 2D and 3D, respectively), shows that to obtain a reasonable and feasible time-scale for simulations, it is essential to very carefully choose the resolution (which can cause simulations to run either much faster or much slower than other CSM implementations), with increasing cell numbers inflicting much less a computational time cost.

### One-to-one mapping between adhesion energies **J** and target perimeter **P**

An important insight that follows from the mathematical analysis is that all possible dynamics within a CSM model can be perfectly captured by two descriptors only, which is significantly smaller than the number of parameters defining the model itself. Because of this, a degenerate mapping can be derived for different sets of adhesion energies (*J*) and perimeter constraints (*P*) that *perfectly* conserve the dynamics (i.e. not only in the limit close to target radius, not only for circular cells, but for any possible configuration). This result is highly relevant, since it has been claimed in the literature that the dynamics for negative *J*-values would be fundamentally different from simulations which use positive *J*-values [22]. We here show, both analytically and through simulations, that a simple scaling allows for using positive or negative values (or a mixture of both) without affecting the dynamics. The energy description for each individual cell *σ* in a tissue, neighbouring one or more cells *σ*^′^ and/or medium *M* can be written as follows:

(40) $$ \begin{aligned} E_{\sigma}=&\;\frac{\sum_{\sigma'}J_{\tau(\sigma),\tau(\sigma')}p_{\sigma,\sigma'}}{2}+J_{\tau(\sigma),M} p_{\sigma,M}+\lambda_{p}\left(p_{\sigma}-P_{\sigma}\right)^{2}\\&+\lambda_{a}\left(a_{\sigma}-A_{\sigma}\right)^{2}\,. \end{aligned}  $$

The term $\sum _{\sigma }J_{\tau (\sigma),\tau (\sigma ')}p_{\sigma,\sigma '}\phantom {\dot {i}\!}$ depicts the total tension associated with all the adhesion contacts between the cell *σ* and the neighbours *σ*^′^, while the term *J*_*τ*(*σ*),*M*_*p*_*σ*,*M*_ depicts the tension with the medium. The division by two captures that the energy linked to the tension between cells is shared by both cells, while this not the case for the tension with the medium [19]. Note that this division simply represents the fact that all interfacial energy contributions are only counted once. The adhesion energies $J_{\tau (\sigma),\tau (\sigma ^{\prime })}\phantom {\dot {i}\!}$ and *J*_*τ*(*σ*),*M*_ are assigned to the contacts between the two cell types *τ*(*σ*) and *τ*(*σ*^′^), and between the cell type *τ*(*σ*) and the medium *M*, respectively. Similarly, the variables $p_{\sigma,\sigma ^{\prime }}\phantom {\dot {i}\!}$ and *p*_*σ*,*M*_ denote the contact length between cells *σ* and *σ*^′^, and between cell *σ* and the medium *M*, respectively. So the total perimeter of the cell *σ* is $p_{\sigma }=p_{\sigma,M}+\sum _{\sigma '}p_{\sigma,\sigma '}$. All the other variables and parameters retain the same meaning as described before for Eq. 1. We first define an energy description with modified *J*- and *P*-values, after which we require both descriptions to be equivalent (except for a possible vertical offset in the energy landscape, which does not affect the dynamics). With *Δ* offsets to the parameters, the energy description looks as follows:

(41) $$ \begin{aligned} E_{\sigma}^{'}=&\;\frac{\sum_{\sigma'}\left(J_{\tau(\sigma),\tau(\sigma')}+\Delta J_{\tau(\sigma),\tau(\sigma')}\right)p_{\sigma,\sigma'}}{2}\\ &+\left(J_{\tau(\sigma),M}+\Delta J_{\tau(\sigma),M}\right)p_{\sigma,M}\\ &+\lambda_{p}\left(p_{\sigma}-\left(P_{\sigma} +\Delta P_{\sigma}\right)\right)^{2}+\lambda_{a}\left(a_{\sigma}-A_{\sigma}\right)^{2}\,. \end{aligned}  $$

The equation presented above can be expanded into

(42) $$  \begin{aligned} E_{\sigma}^{'}=&\;\frac{\sum_{\sigma'}J_{\tau(\sigma),\tau(\sigma')}p_{\sigma,\sigma'}}{2}+J_{\tau(\sigma),M}p_{\sigma,M}+\lambda_{p}\left(p_{\sigma}-P_{\sigma}\right)^{2}\\ &+\lambda_{a}\left(a_{\sigma}-A_{\sigma}\right)^{2}+\frac{\sum_{\sigma'}\Delta J_{\tau(\sigma),\tau(\sigma')}p_{\sigma,\sigma'}}{2}\\&+\Delta J_{\tau(\sigma),M}p_{\sigma,M}-2\lambda_{p}p_{\sigma}\Delta P_{\sigma}+2\lambda_{p}P_{\sigma}\Delta P_{\sigma}\\&+\lambda_{p}\Delta P_{\sigma}^{2}\,, \end{aligned}  $$

which is simply

(43) $$ \begin{aligned} E_{\sigma}^{'}=&\;E_{\sigma}+\frac{\sum_{\sigma'}\Delta J_{\tau(\sigma),\tau(\sigma')}p_{\sigma,\sigma'}}{2}+\Delta J_{\tau(\sigma),M}p_{\sigma,M}\\&-2\lambda_{p}p_{\sigma}\Delta P_{\sigma}+2\lambda_{p}P_{\sigma}\Delta P_{\sigma}+\lambda_{p}\Delta P_{\sigma}^{2}\,. \end{aligned}  $$

Because the dynamics of the system described by *Eσ*′ will not be different from *E*_*σ*_ if $E{}_{\sigma }^{'}=E_{\sigma }+\text {const.},$ it is easily recognizable that the terms $2\lambda _{p}P_{\sigma }\Delta P_{\sigma }+\lambda _{p}\Delta P_{\sigma }^{2}$, which do not depend on any variable, do not contribute at all to the dynamics. Therefore, in order for the dynamics of both systems to be equal, the remaining terms $\frac {1}{2}\sum _{\sigma '}\Delta J_{\tau (\sigma),\tau (\sigma ')}p_{\sigma,\sigma '}+\Delta J_{\tau (\sigma),M}p_{\sigma,M}-2\lambda _{p}p_{\sigma }\Delta P_{\sigma }$ will have to vanish:

(44) $$ \frac{\sum_{\sigma'}\Delta J_{\tau(\sigma),\tau(\sigma')}p_{\sigma,\sigma'}}{2}+\Delta J_{\tau(\sigma),M}p_{\sigma,M}-2\lambda_{p}p_{\sigma}\Delta P_{\sigma}=0\,.  $$

One can find now what *ΔP*_*σ*_ should become to compensate for the change in the adhesion-energies $\Delta J_{\tau (\sigma),\tau (\sigma ^{\prime })}\phantom {\dot {i}\!}$:

(45) $$ \Delta P_{\sigma}=\frac{\sum_{\sigma}\Delta J_{\tau(\sigma),\tau(\sigma')}p_{\sigma,\sigma'}+2\Delta J_{\tau(\sigma),M}p_{\sigma,M}}{4\lambda_{p}p_{\sigma}}\,,  $$

which, put in a more simplified form, gives:

(46) $$ \Delta P_{\sigma}=\frac{\overline{\Delta J_{w}}}{2\lambda_{p}}\,,  $$

where $\overline {\Delta J_{w}}\,=\,\left (\frac {1}{2}\sum _{\sigma '}\Delta J_{\tau (\sigma),\tau (\sigma ')}p_{\sigma,\sigma '}+\Delta J_{\tau (\sigma),M}p_{\sigma,M}\right)/p_{\sigma }$ is the weighted mean adhesion-driven interfacial tension. This simple expression shows the balance that has to exist to keep the dynamics the same if the adhesion energies are to be changed, negative- or positive-wise. The equation shows that in general this compensation depends on the extent of the contact length of cell *σ* with other neighbouring cells, i.e. $p_{\sigma,\sigma ^{\prime }}$ (this dependence is implicit in $\overline {\Delta J_{w}}$), and it is therefore not possible to find a unique mapping between a change in one specific adhesion energy $\phantom {\dot {i}\!}J_{\tau (\sigma),\tau (\sigma ^{\prime })}$ and the perimeter constraints *P*_*σ*_. The reason is that if one changes a specific adhesion energy by adding $\Delta J_{\tau (\sigma),\tau (\sigma ^{\prime })}\phantom {\dot {i}\!}$ ($J_{\tau (\sigma),\tau (\sigma ^{\prime })}\to J_{\tau (\sigma),\tau (\sigma ')}+\Delta J_{\tau (\sigma),\tau (\sigma ')}\phantom {\dot {i}\!}$), the equilibrium contact lengths may change for that cell. This changes the weights (of the weighted average $\overline {\Delta J_{w}}$), i.e. $p_{\sigma,\sigma ^{\prime }}\phantom {\dot {i}\!}$, and probably also the cell shape, as the cell will now have a new equilibrium partitioning of its contact lengths with the neighbouring cells.

In contrast, in the specific case that all adhesion energies are changed concomitantly there is a perfect one-to-one mapping between the change in adhesion and the change in perimeter constraint. By choosing $\Delta J_{\tau (\sigma),\tau (\sigma ^{\prime })}=\Delta J_{cell,cell}\phantom {\dot {i}\!}$ to be equal for all *σ*, *σ*^′^, and $\Delta J_{\tau (\sigma),M}=\Delta J_{cell,medium}=\frac {1}{2}\Delta J_{cell,cell}$ to be equal for all *σ*, Eq. 45 can be rewritten as

(47) $$ \Delta P_{\sigma}=\frac{\frac{1}{2}\Delta J_{cell,cell}\left(\sum_{\sigma}\Delta p_{\sigma,\sigma'}+p_{\sigma,M}\right)}{2\lambda_{p}p_{\sigma}}=\frac{\Delta J_{cell,cell}}{4\lambda_{p}}\,.  $$

**Figure 6:**
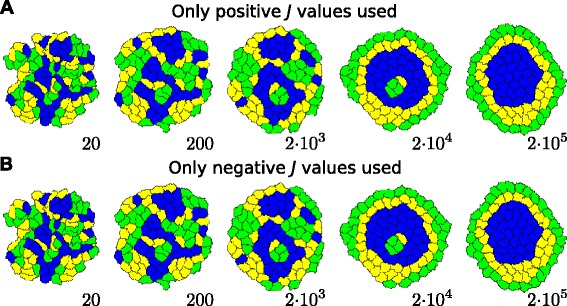
One-to-one mapping between adhesion energy *J* and target perimeter *P*.**(A)** Simulation using positive *J*-values only, showing cell sorting of three different cell types, G(reen), Y(ellow), and B(lue), in a tissue surrounded by M(edium). Parameters used are *J*_*G*,*G*_=*J*_*Y*,*Y*_=*J*_*B*,*B*_=400, *J*_*G*,*M*_=600, *J*_*Y*,*M*_=1,200, *J*_*B*,*M*_=1,800, *J*_*G*,*Y*_=*J*_*Y*,*B*_=800, *J*_*G*,*B*_=1,400; *A*=30; *P*=67; *λ*_*a*_=1,000; *λ*_*p*_=20; and *T*=600. **(B)** Simulation using negative *J*-values only, presenting *exactly* the same dynamics as the simulation shown in **(A)**. (Note that the same initial conditions and random seed were used.) Parameters are the same as in **(A)**, except for *J*_*G*,*G*_=*J*_*Y*,*Y*_=*J*_*B*,*B*_=−3,200, *J*_*G*,*M*_=−1,200, *J*_*Y*,*M*_=−600, *J*_*B*,*M*_=0, *J*_*G*,*Y*_=*J*_*Y*,*B*_=−2,800, *J*_*G*,*B*_=−2,200; and *P*=22. Here, only the CPM parameters are given, which can be translated into the actual values using Eq. 31.

In short, increasing or decreasing all cell-cell adhesion energies with a constant *ΔJ*_*cell*,*cell*_ (and all cell-medium adhesion energies with half of this value) can be perfectly compensated by concurrently increasing or decreasing all perimeter constraints with the same value divided by 4*λ*_*p*_. CPM simulations confirm this one-to-one mapping between the change *ΔJ* and the corresponding change *ΔP*_*σ*_ needed to keep the dynamics the same (Figure 6). Importantly, even in the case that *ΔJ* is sufficiently negative to change all initially positive *J*-values into negative *J*-values, the dynamics remain unaffected (Figure 6B). In 3D the same correspondence holds, provided the obvious changes in notation are made, so the relationship is

(48) $$ \Delta S_{\sigma}=\frac{\overline{\Delta J_{w}}}{2\lambda_{s}}  $$

for the general case, and

(49) $$ \Delta S_{\sigma}=\frac{\Delta J_{cell,cell}}{4\lambda_{s}}  $$

for the specific case of a concurrent change of all *J*-values with *ΔJ*_*cell*,*cell*_ (but cell-medium adhesion energies with half of this value).

When applying this method, it is important to realize that there is no such scaling possible for the parameter *λ*_*p*_. One consequence is that one cannot rescale a model which does not take a perimeter constraint into account (*λ*_*p*_=0) into one which does. That is, the rescaling only works once the perimeter constraint is already taken into account. Likewise, to be explicit, rescaling the model such that *P*_*σ*_=0 does not mean that perimeter constraint has been taken out of the equation. It rather implies a perimeter constraint with a rest length of zero.

### Single cell simulations

**Figure 7:**
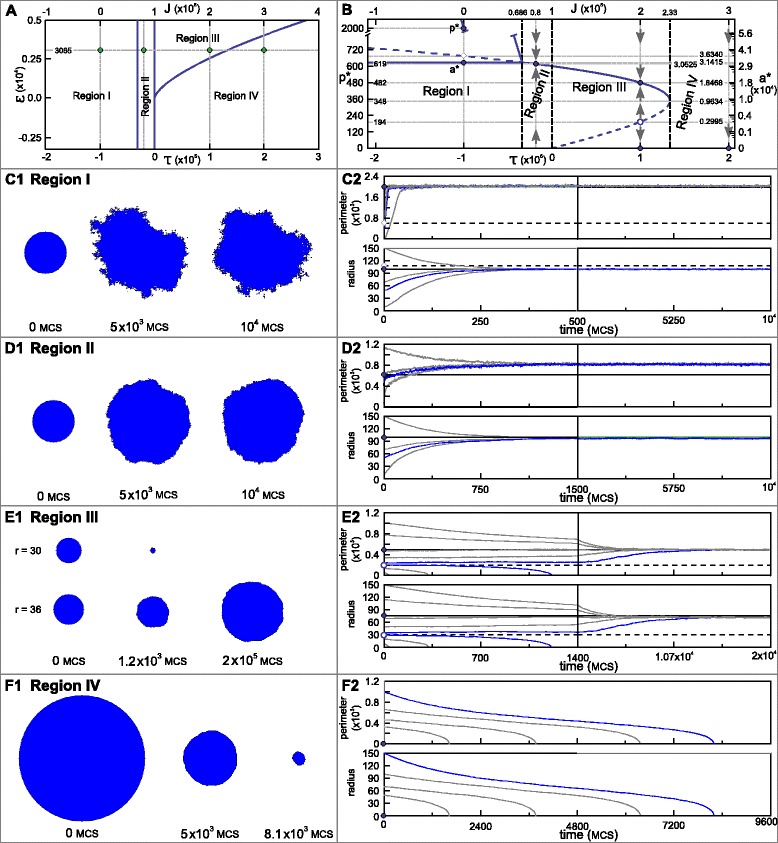
CPM simulations of individual cells within the four parameter regions.**(A)** Parameter settings at which the simulations are performed. In all simulations, *ε*=3,065. In regions I–IV, *τ*=−10^5^, −2·10^4^, 10^5^, and 2×10^5^, respectively. **(B)** Analytically derived bifurcation diagram of the cell dynamics as a function of *τ* (or equivalently *J*), with *ε* fixed at 3,065. Both equilibrium perimeter *p* ^∗^ and area *a* ^∗^ are shown. For *J*>68,584 (see Eq. 14b) cells take up a circular shape, so *p* ^∗^ and *a* ^∗^ are trivially related; for *J*<68,584, *p* ^∗^ and *a* ^∗^ vary independently of each other. Solid and dashed lines represent stable and unstable equilibria, respectively. Dots refer to the equilibria at simulation parameter settings, which can be stable (blue circles), unstable with respect to size (open circles), or unstable with respect to shape (open diamonds). Boundaries between the four parameter regions are defined by black dashed lines. (Note the discontinuity in the *p* ^∗^-axis.) **(C1**–**F1)** Snapshots of single cell simulations in different regions: Region I with initial radius *r*=50**(C1)**; Region II with initial *r*=50**(D1)**; region III with initial *r*={30, 36}**(E1)**; Region IV with initial *r*=150**(F1)**. **(C2**–**F2)** Time evolution of the perimeter (top) and radius (bottom) of simulated cells for different initial conditions with variable initial cell radius. Blue line(s) correspond to the individual cell dynamics shown in the snapshots **(C1**–**F1)**. Black solid lines indicate the analytically derived stable equilibria, dashed lines the unstable equilibria. Further parameter details are given in Appendix C.

Having discussed fundamental issues regarding anisotropy, perimeter and scaling, we next study whether the analytical results can be corroborated by CPM simulations. To illustrate all predicted qualitatively distinct dynamics, we performed 2D single cell CPM simulations for each of the four described regions. Given that the type of dynamics is only dependent on two aggregate parameters, *τ* and *ε*, we arbitrarily defined four sets of parameters, one for each of those regions, indicated in Figure 7A. The expected equilibrium area *a*^∗^ and perimeter *p*^∗^ are shown in Figure 7B.

In region I, a cell of initial radius 50*Δx* grows rapidly in size (Figure 7C1). In this region, the cell is expected to have a negative interfacial tension at the circular shape. Therefore, the circular shape is unstable and the area and perimeter constraints can be both fulfilled independently, while the cell takes up a complex shape. In such a case, the stable equilibrium area *a*^∗^ is directly defined by the cell’s target area (*a*^∗^=*A*), while the equilibrium perimeter *p*^∗^ is determined by an interfacial tension of *γ*=0 ($p^{*}=-\frac {\tau }{2\lambda _{p}}=P-\frac {J}{2\lambda _{p}}$). Figure 7C2 shows the time dynamics of the perimeter and radius of four cells with different initial sizes, the blue line indicating the cell (Figure 7C1). The dashed lines indicate the expected perimeter and radius at the unstable equilibrium with circular cell shape, while the solid lines indicate the expected perimeter and radius when both constraints are fulfilled independently in a complex cell shape. After 500 Monte Carlo Step (MCS), the predicted *p*^∗^=2000 *Δx* and *a*^∗^=31416*Δx*^2^ (or *r*^∗^=100*Δx*) perfectly match the simulations, while the cell shapes are clearly deviating from being circular.

In region II, the simulated cell shows a more isotropic shape that approaches a circular shape (Figure 7D1). The simulated cells starting at different radii all converge to the same equilibrium perimeter and area (Figure 7D2). While the analytical equilibrium area *a*^∗^ is approached very closely by the numerical simulations, the analytical equilibrium perimeter *p*^∗^=619*Δx* deviates considerably from what is observed in the simulations (which is around 800*Δx*, see Figure 7D2). This deviation can be explained by the membrane fluctuations that are due to the stochastic nature of the CPM update scheme. Close to region I the interfacial tension at equilibrium is only marginally larger than zero. Consequently, stochasticity can easily cause the formation of excess perimeter, causing its length to deviate from the analytical, deterministic prediction. This effect strongly depends on the simulation temperature parameter *T*. When this parameter is lowered, the difference between the predicted and numerical observed perimeter at equilibrium is reduced. Note, however, that low simulation temperatures can also introduce undesirable grid effects [35].

In region III, the cell dynamics exhibits bistability. In Figure 7E2, the dashed lines indicate the analytical unstable equilibrium (*p*^∗^=194*Δx*, *a*^∗^=2995*Δx*^2^) and the solid lines the stable non-trivial equilibrium (*p*^∗^=482*Δx*, *a*^∗^=18468*Δx*^2^), with the other stable equilibrium given by *p*^∗^=0*Δx*, *a*^∗^=0*Δx*^2^. Figure 7E1 shows the simulations of two different cells, one starting below the unstable steady-state (initial radius *r*=30*Δx*), the other starting above the unstable equilibrium (initial radius *r*=36*Δx*). While the larger cell increases in size, the smaller cell eventually disappears. Note that in this region there is hardly any discrepancy between the analytical and observed values, due to a much higher interfacial tension at equilibrium.

In region IV, the simulated cells exhibit a very smooth membrane, due to an even higher interfacial tension. The interfacial tension dominates over the cell’s pressure at any cell size, triggering a continuous size decrease until the cell vanishes (Figure 7F1). Both the perimeter and area concomitantly decrease until the trivial equilibrium *p*^∗^=0*Δx*, *a*^∗^=0*Δx*^2^ is reached (Figure 7F2).

**Figure 8:**
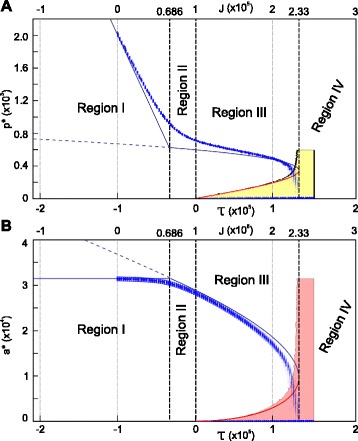
Comparison between the analytically derived equilibria and the equilibria observed in CPM simulations. Shown are the analytical predictions of the equilibrium perimeter **(A)** and equilibrium area **(B)** as a function of *τ*, together with the numerically observed equilibrium distributions. (Parameters were chosen in such a way that *ε*>0, which ensures that varying only *τ* allows for passing through all four qualitatively different regions.) Solid blue lines indicate predicted stable equilibria, solid red lines predicted unstable equilibria with respect to size, and dashed blue lines unstable equilibria with respect to shape. Dots correspond to the numerical results. The mean and standard deviation for each *τ*-value were calculated by averaging over the last 1,000 MCS of 100 independent simulations. The intensity of the dots indicates the fraction of cells not disappearing. The yellow and pink area indicate the combinations of *τ*-value and initial perimeter and area for which in more than 50*%* of the simulations the cells disappeared. All simulations used a 6th level neighbourhood and *R*_*a*_=100 (*A*=31,416); *P*=2,000 ($P_{_{\mathit {CPM}}}=36,000$); *λ*_*a*_=0.0625; *λ*_*p*_=25 ($\lambda _{p_{\,\mathit {CPM}}}=0.07716$); and *T*=20,000. The *J* parameter was swept over the interval [ 0, 2.5·10^5^] ($J_{_{\mathit {CPM}}}$ over [ 0, 13,889]).

To further determine the level of agreement between the CPM simulations and the mathematical analysis, we performed 10,000 simulations of a single cell, varying the *J*-value as well as the initial radius of the cell. From the analysis, we know that cells should either shrink to a zero size (in region IV, as well as too small cells in region III), or should reach an equilibrium area and perimeter. We therefore registered after 10,000 MCS whether the cell had disappeared, and, if not, what its size and perimeter had become (averaged over the last 1,000 MCS). We then plotted those measurements on top of the analytically predicted bifurcation diagram, using the corresponding *τ*-value as the bifurcation parameter (Figure 8), in the following way. For each cell that had disappeared, we plotted the *initial* perimeter and area (yellow and pink regions, respectively). The boundary between initial sizes that do or do not lead to shrinkage should correspond to the unstable equilibrium in region III (solid red line), while in region IV cells should always disappear. For each cell that did not disappear, we plotted the mean and standard deviation of the *final* perimeter and area (blue dots, intensity indicates fraction of cells not disappearing). Those points should correspond to the stable equilibrium in region I, II and III (solid blue line). We indeed find a close correspondence between the analytical and computational results (Figure 8), but with some remarkable differences. First, we find that region IV, in which cells are never sustained, starts at slightly lower *τ*-values in the numerical simulations, and that the stable equilibrium in region III lies at slightly lower values and the unstable equilibrium at slightly higher area values. Both are due to the fact that, while the analysis was done for a perfectly round cell, such a shape is not actually obtained in the CPM. This stems from the stochastic fluctuations that form an intrinsic part of the CPM, rather than from the fact that the CPM is lattice-based (as discussed before, this only causes a small deviation, especially when using 6th level neighbourhood, as done here). Those fluctuations cause the cell to temporarily deviate from a round shape, increasing the amount of perimeter per area. As will be discussed in the next section, this causes small changes in the position of the bifurcation lines, as well as in the equilibrium area and perimeter.

Secondly, in the analysis we find a sharp transition from region I (with a negative interfacial tension for an equilibrium circular cell, leading to a non-circular cell shape) and region II (with a positive interfacial tension, in which a circular shape is preferred). In the computer simulations we find that this transition is more blurred: when the interfacial tension becomes small, the forces restoring a round shape from thermally-driven shape deformations become small as well. Hence, close to the region I/II boundary the cell shape increasingly deviates from a round shape, resulting in a smooth transition. Still, overall, those simulations present a close match between the expected perimeter and area from the mathematical analysis. It illustrates that the CPM, albeit a lattice-based, discrete and stochastic model formalism, still presents equilibrium dynamics as expected from the basics of cell surface mechanics.

**Figure 9:**
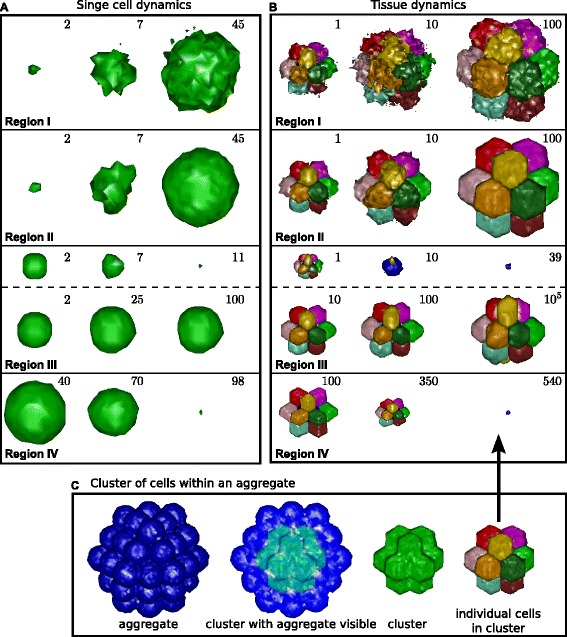
3D CPM simulations of single cells and tissues, within the four qualitatively different regions.**(A)** Single cell dynamics. All simulations used a 12th level neighbourhood ($\xi =\frac {169}{4}\pi \approx 132.73$) and *V*=5,000 (*R*_*v*_=10.6); *S*=500 ($S_{_{\mathit {CPM}}}=66,366$); *λ*_*v*_=50; *λ*_*s*_=25 ($\lambda _{s_{\,\mathit {CPM}}}=0.001419$); and *T*=10,000. The *J*-value was modified to set *τ*: In region I, *J*=−55,000 ($J_{_{\mathit {CPM}}}=-414$); in region II, *J*=20,000 ($J_{_{\mathit {CPM}}}=150$); in region III, *J*=1,000,000 ($J_{_{\mathit {CPM}}}=7,533$); and in region IV, *J*=1,400,000 ($J_{_{\mathit {CPM}}}=10,547$). Initial radii are: region I and II, *r*=1; region III, top, *r*=4; region III, bottom, *r*=5; region IV, *r*=30. **(B)** Tissue dynamics. Shown are 13 individual cells located within a larger aggregate of cells **(C)**. All parameters are the same as in **(A)**, except for the *J*-values to set *τ* to be within the right zones (since the zone boundaries are different for spherical and rhombic dodecahedronal cell shapes). Note that $J_{_{\mathit {CPM}}}=J_{C,M}=2J_{C,C}$, capturing the fact that in the CPM formalism the contribution by *J* to the interfacial tension along cell-cell boundaries — but not cell-medium boundaries — is shared between neighbouring cells. In region I, *J*=−65,000 ($J_{_{\mathit {CPM}}}=-489$); in region II, *J*=20,000 ($J_{_{\mathit {CPM}}}=150$); in region III, *J*=900,000 ($J_{_{\mathit {CPM}}}=6,780$); and in region IV, *J*=1,400,000 ($J_{_{\mathit {CPM}}}=10,547$). Initial radii are: region I and II, *r*=5; region III, top, *r*=3; region III, bottom, *r*=5; region IV, *r*=20.

Likewise, the analytical 3D bifurcation diagram (Figure 3B) correctly predicts the single cell dynamics in 3D CPM simulations (Figure 9A), although again small deviations are observed close to region boundaries (data not shown).

### Epithelial cell packing

In the previous section we have pointed out deviations from the analytical results when cell shapes differ too much from a perfect circle (in 2D) or perfect sphere (in 3D). Clearly, within a packed tissue context such an assumption of circularity is invalid. We therefore questioned whether the analysis could be extended to non-circular shapes.

For 2D we focus on epithelial tissue. Simple epithelium is normally a cell monolayer tissue made of cells strongly adhering to each other in a plane of cell junctions. The cellular organization of the epithelium derives from the mechanical properties, particularly at the level of cell junctions. Several modelling studies have looked whether the morphological properties of epithelial tissue can be derived from simple rules describing its mechanical properties [24,25]. Here, we try to approach the characterization of epithelial cell packing using the CSM description. We explore the assumption that the behaviour of an epithelial sheet is no more than the collective behaviour of its individual cells. Due to high cell density within epithelial tissues, cells are typically densely packed and exhibit hexagonal shapes. We therefore seek how a cell behaves when it is constrained to a hexagonal shape.

Du et al. [28] numerically solved the energy function for an infinite system of hexagonal cells. Instead of writing the energy function for the hexagonal cell, we rather consider a broader class of shapes, which includes the hexagon, but also the circle, through a parametrisation which links the amount of perimeter to the amount of surface area. We do so by choosing for any cell shape an arbitrary length factor *l* (which could be the longest radius, the length of a rib or any other convenient choice), and then introducing the constants *k*_*p*_ and *k*_*a*_, which scale the length factor *l* to the perimeter *p* and area *a*, respectively:

(50a) $$\begin{array}{*{20}l} p &= k_{p}l\,, \end{array} $$

(50b) $$\begin{array}{*{20}l} a &= k_{a}l^{2}\,. \end{array} $$

For example, a hexagonal cell can be described by *k*_*p*_=6 and $k_{a}=\frac {3\sqrt {3}}{2}$, which can be derived when taking for *l* the polygon’s side length (but any choice for *l* will lead to the same result). Likewise, a circular cell can be described by *k*_*p*_=2*π* and *k*_*a*_=*π*, typically taking for *l* the radius *r*.

The energy *E* as function of *l* reads as

(51) $$\begin{array}{*{20}l} &E\left(\left.l\right|J,\lambda_{p},L_{p},\lambda_{a},L_{a},k_{p},k_{a}\right)={Jk}_{p}l+\lambda_{p}\left(k_{p}l-k_{p}L_{p}\right)^{2}\\ &\quad+\lambda_{a}\left(k_{a}l^{2}-k_{a}{L_{a}^{2}}\right)^{2}\,. \end{array} $$

While the parameters *k*_*p*_ and *k*_*a*_ are constants that relate the perimeter and area with *l*, *L*_*p*_ and *L*_*a*_ are the reciprocal target lengths of the target perimeter *P* and area *A*.

The analysis from the single cell case can be easily performed for this more general case, see Appendix B, yielding

(52a) $$\begin{array}{*{20}l} \frac{\partial E}{\partial l} &= k_{p}\left(\gamma-2\frac{k_{a}}{k_{p}}l\Pi\right)\,, \end{array} $$

(52b) $$\begin{array}{*{20}l} \gamma &= J+2k_{p}\lambda_{p}\left(l-L_{p}\right)\,, \end{array} $$

(52c) $$\begin{array}{*{20}l} \Pi &= -2k_{a}\lambda_{a}\left(l^{2}-{L_{a}^{2}}\right)\,, \end{array} $$

(52d) $$\begin{array}{*{20}l} \tau &= J-2k_{p}\lambda_{p}L_{p}\,, \end{array} $$

(52e) $$\begin{array}{*{20}l} \epsilon &= \frac{{L_{a}^{2}}}{3}-\frac{{k_{p}^{2}}\lambda_{p}}{6{k_{a}^{2}}\lambda_{a}}\,, \end{array} $$

(52f) $$\begin{array}{*{20}l} \gamma\left(l^{*}\right)=0 & \Rightarrow \tau=-2k_{p}\lambda_{p}L_{a}\,, \end{array} $$

(52g) $$\begin{array}{*{20}l} \left.\frac{\partial E}{\partial l}\right|_{l=0}=0 & \Rightarrow \tau=0\,, \end{array} $$

(52h) $$\begin{array}{*{20}l} \left.\frac{\partial^{2}E}{\partial l^{2}}\right|_{l=l^{*}}=0 & \Rightarrow \tau=\frac{8{k_{a}^{2}}\lambda_{a}}{k_{p}}\epsilon^{\frac{3}{2}}\,. \end{array} $$

**Figure 10:**
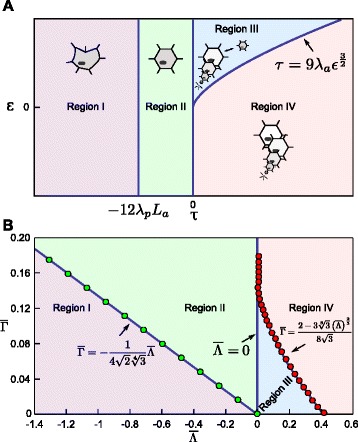
The biophysical parameter space in the context of 2D cell-packing.**(A)** Biophysical parameter space for the hexagonal cell following *p*=*k*_*p*_ *l* and *a*=*k*_*a*_ *l* ^2^, with *k*_*p*_=6 and $k_{a}=\frac {3\sqrt {3}}{2}$. **(B)** Biophysical parameter space for the hexagonal cell in terms of $\overline {\Lambda }$ and $\overline {\Gamma }$, adopting the notation of Farhadifar et al. [25]. Blue lines depict the analytically derived bifurcation lines, red and green circles are extracted through image analysis of Figure 1 in [25]. The green dots perfectly match the bifurcation line $\overline {\Gamma }=-\frac {1}{4\sqrt {2}\sqrt [4]{3}}\overline {\Lambda }$; likewise the red dots perfectly match the bifurcation line $\overline {\Gamma }=\frac {2-3\sqrt [6]{3}\left (\overline {\Lambda }\right)^{\frac {2}{3}}}{8\sqrt {3}}$. We predict one more bifurcation line, $\overline {\Lambda }=0$, while in [25] region II and III are considered a single region, the so called “Hexagonal Network”.

The analysis shows that not only for the circular shape but for any choice of specific cell shape, all possible dynamics within the class of CSM models can be fully captured by a two-parametric bifurcation diagram, defined by the aggregate parameters *τ* and *ε* and determined by its perimeter/area balance. Due to the generalization, *τ* and *ε*, as well as the bifurcation lines between region I and II (*γ*(*l*^∗^)=0) and between III and IV ($\left.\frac {\partial ^{2}E}{\partial l^{2}}\right |_{l=l^{*}}=0$) become a function of the perimeter/area balance itself. The overall structure of the bifurcation diagram, however, does not change; the same qualitative results as obtained for the circular cell hold for any other cell shape, including the hexagonal shape. Figure 10A shows the bifurcation diagram specifically for the hexagonal case. Comparing it with Figure 3A indeed shows that the specific parameter values for the bifurcations change, but the overall structure does not.

We next determined whether this analytical result can indeed correctly describe cell behaviour of hexagonal cells in a multicellular context. We therefore compared the analytical predictions to published simulation results of a vertex model [25], as well as to a series of tissue simulations using the CPM formalism.

#### Vertex model cell packing simulations

To show that the results regarding the epithelial cell packing are a general consequence of the CSM model and not a quality particular to the CPM or any other numerical implementation, we took published data about the modelling of epithelial cell packing that uses a different mathematical formalism and compared it to our analytical predictions. For this, we used the work reported in Farhadifar et al. [25], where the authors used the Vertex model to investigate the mechanical properties of epithelial cell packing. Their approach describes the CSM tensions using the same energy description as presented here, compare Eq. 1 with Box 1 in [25].

To compare with the results from [25], we introduce the correspondence between their notation and ours: *J*=*Λ*, *λ*_*p*_=*Γ*, *λ*_*a*_=*K*, *A*=*A*^0^ and *P*=0. The fact that they assumed *P*=0 in their approach means that our description of the CSM holds more generally, because with such an assumption the influence of the cortical tension on the interfacial tension is always positive. The authors also introduced a normalised tension $\overline {\Lambda }$ and normalised contractility $\overline {\Gamma }$ (see Eq. 84), which were used to depict their biophysical parameter space.

The derivation of the conditions defining regions I–IV for this parametrization is straightforward from Eq. 52f, Eq. 52g and Eq. 52h, and is given in Appendix D. From the condition *γ*(*l*^∗^)>0 one derives $\overline {\Gamma }>-\frac {\sqrt {k_{a}}}{2k_{p}}\overline {\Lambda }$; the condition $\left.\frac {\partial E}{\partial l}\right |_{l=0}=0$ simply yields $\overline {\Lambda }=0$; and the condition $\left.\frac {\partial ^{2}E}{\partial l^{2}}\right |_{l=l^{*}}=0$ gives $\overline {\Gamma }=\frac {4k_{a}-3\left (k_{a}k_{p}\overline {\Lambda }\right)^{\frac {2}{3}}}{2{k_{p}^{2}}}$. As is shown in Figure 10B, for the particular case of the hexagon, for which *k*_*p*_=6 and $k_{a}=\frac {3\sqrt {3}}{2}$, the three bifurcation lines become:

(53a) $$\begin{array}{*{20}l} \overline{\Gamma} & = -\frac{1}{4\sqrt{2}\sqrt[4]{3}}\overline{\Lambda}\,, \end{array} $$

(53b) $$\begin{array}{*{20}l} \overline{\Lambda} & = 0\,, \end{array} $$

(53c) $$\begin{array}{*{20}l} \overline{\Gamma} & = \frac{2-3\sqrt[6]{3}\left(\overline{\Lambda}\right)^{\frac{2}{3}}}{8\sqrt{3}}\,. \end{array} $$

By representing the bifurcation diagram of Figure 10A in the equivalent ($\overline {\Lambda },\overline {\Gamma }$)-space, we show that the analytical results are perfectly matched by the Vertex model simulations of [25] (Figure 10B): Eq. 53a, which separates the positive from the negative interfacial tension regime, matches the boundary obtained by simulation (green dots in Figure 10B, extracted from [25]). Eq. 53b, which separates the regime with a single positive stable equilibrium from the bistable regime in our analysis, is not present in their simulation bifurcation diagram. Nevertheless, when we follow our analysis, the so-called Case II of their parameter space should be located within the bistable regime. Indeed, a snapshot of their simulation reveals a huge variation in cell size, strongly suggesting bistability (see Figure 2H in [25]). Finally, Eq. 53c, which separates region III and IV, lies right on top of their numerically-derived line that separates the “Hexagonal network” and “cell vanishing” regimes. Please note that a comparable analytical effort has been published to understand the boundaries between the different observed behaviours [30]. That study, however, did not note the distinction between region II and III, and they derived an approximation of the boundary between region III and IV, while the solution presented here is exact. Consequently, unlike [30], our analytically derived boundary between region III and IV matches perfectly the numerical simulations over their full domain.

These results show that the tissue properties which were defined in [25] as “Soft Network”, “Hexagonal Network” and “Cell Vanishing” perfectly correspond to region I, region II/III, and region IV, respectively, as presented in this paper. It highlights that such tissue properties are basically a manifestation of the mechanical properties of individual cells and not due to collective cell behaviour *per se* (except that collective cell behaviour is needed to give rise to the hexagonal cell shapes). In fact, knowledge on the expected cell shape combined with single cell analysis turned out to be sufficient to account for the whole range of observed tissue behaviour.

#### CPM cell packing simulations

**Figure 11:**
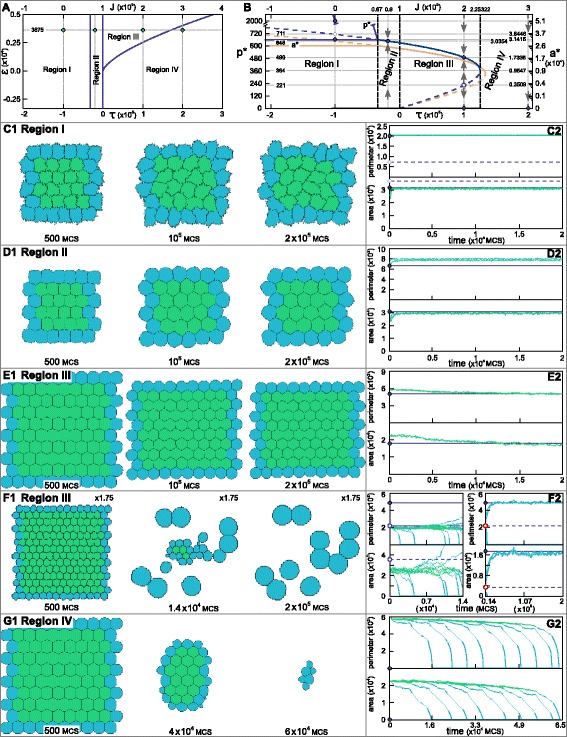
CPM simulations of epithelial packing within the four parameter regions.**(A)** Parameter settings at which the simulations are performed. In all simulations, *ε*=3,675. In regions I–IV, *τ*=−10^5^, −2·10^4^, 10^5^, and 2·10^5^, respectively. **(B)** Blue lines: Analytically derived bifurcation diagram of the cell dynamics, assuming hexagonal cell shape, as a function of *τ* (or equivalently *J*), with *ε* fixed at 3,675. Orange lines: corresponding bifurcation diagram for circular cell shape. Both equilibrium perimeter *p* ^∗^ and area *a* ^∗^ are shown. For *J*>67,011 cells take up a hexagonal shape, so *p* ^∗^ and *a* ^∗^ are trivially related; for *J*<67,011, *p* ^∗^ and *a* ^∗^ vary independently. Solid and dashed lines represent stable and unstable equilibria, respectively. Dots refer to the equilibria at simulation parameter settings, which can be stable (blue circles), unstable with respect to size (open circles), or unstable with respect to shape (open diamonds). The bifurcation points are indicated by black dashed lines. (Note the discontinuity in the *p* ^∗^-axis.) **(C1**–**G1)** Snapshots of epithelial packing simulations in the different regions, as indicated. Initial cell size in **(E1)** was larger than the unstable equilibrium (*p* ^∗^=221, *a* ^∗^=3,509), whereas in **(F1)** it was smaller. All snapshots are displayed at the same scale, except for **(F1)**, enlarged by a factor 1.75. Hexagonal cells, defined as cells with exactly six neighbours, are coloured green, all others light blue. **(C2**–**G2)** Time evolution of the perimeter (top) and area (bottom) of a subset of individual cells within the simulated epithelial tissue. Green and blue again indicate whether cells have six neighbours. Black solid lines indicate the analytically derived stable equilibria, dashed lines the unstable equilibria. Further parameter details are given in Appendix C.

We then asked if a comparable level of correspondence can be found between the CPM simulations and the mathematical analysis when considering a homogeneous tissue. As for the single cell case, we performed several simulations in order to illustrate and compare the different parameter spaces. Again we defined four sets of parameters, one for each region (Figure 11A). A bifurcation diagram with the expected equilibria in each region, under the assumption of a hexagonal cell shape, is shown in Figure 11B. (For comparison, the equilibria for the circular case are shown as well.)

In region I the cells in the cluster show a fringed border (Figure 11C1). In this region, the cells are expected to have a negative interfacial tension when taking up a hexagonal shape. Consequently, the hexagonal shape is unstable, and the tissue is formed by dynamically changing, non-hexagonal cells, with tissue topology alterations by neighbour changes, during which some cells loose their contact, while others come into contact with each other (so-called T1 transitions, see [19]). In this regime, both perimeter and area constraint are fulfilled independently, the equilibrium area *a*^∗^ defined by the cell’s target area and the equilibrium perimeter *p*^∗^ determined by a zero cell interfacial tension, as shown in Figure 11B, C2. The predicted *p*^∗^=2,000*Δx* and *a*^∗^=31,416*Δx*^2^ (or *l*^∗^=110 *Δx*) are perfectly matched by the simulations.

In region II, the simulated cell cluster takes up a honeycomb shape, with each individual cell approaching the expected hexagonal shape (Figure 11D1). After initiation of the simulation, all cells rapidly converge to the same equilibrium perimeter and area (Figure 11D2). As was also observed for the single cell simulations, the observed equilibrium perimeter is larger than the expected equilibrium perimeter *p*^∗^=648*Δx*, due to the stochasticity of the CPM update scheme. For this particular parameter choice the difference is around 20%, but it increases when the equilibrium is followed towards region I and decreases when followed towards region IV, with lower simulation temperatures yielding a much closer match.

In region III, the cells exhibit bistability (Figure 11E1, F1). Figure 11E1 illustrates that when a simulation is initiated with cells that are larger than the unstable equilibrium (*p*^∗^=221*Δx*, *a*^∗^=3,509*Δx*^2^), the cells evolve towards the non-trivial stable equilibrium (*p*^∗^=490*Δx*, *a*^∗^=17,336*Δx*^2^, Figure 11E2). In contrast, in a simulation initiated with cells below the unstable equilibrium (Figure 11F1), most cells vanish (*p*^∗^=0*Δx*, *a*^∗^=0*Δx*^2^), but some, due to stochastic fluctuations, pass the unstable equilibrium to end up in the non-trivial stable one (Figure 11F2). Note that due to the increased interfacial tension compared to the previous simulations, fluctuations that deviate the cell shape from perfect hexagonality are much less pronounced, and hence the simulations match the analytical results more precisely.

In region IV, the cell edges are even smoother due to the high interfacial tension (Figure 11G1). However, all cells vanish rapidly, reaching the trivial equilibrium (*p*^∗^=0*Δx*, *a*^∗^=0*Δx*^2^, Figure 11G2).

Overall, the predicted behaviour for a hexagonal cell closely matches the numerical CPM simulations of multiple cells, illustrating that again the tissue behaviour can be captured by CSM considerations at the cellular level. A complete overview of the parameters used in the 2D analysis, the general results for any cell shape, and the specific ones for circular and hexagonal cells, are given in Table 3.

**Table 3:** **Parameters used in the 2D analysis and their meaning**

**Parameter**	**Meaning**	**General**	**Circle**	**Hexagon**
*E*	Energy function or Hamiltonian	*J* *p*+*λ*_*p*_(*p*−*P*)^2^+*λ*_*a*_(*a*−*A*)^2^		
*J*	Adhesion energy (per contact length)	*J*	*J*	*J*
*p*	Cell perimeter	*k*_*p*_ *l*	2*π* *r*	6*l*
*a*	Cell area	*k*_*a*_ *l* ^2^	*π* *r* ^2^	$\frac {3\sqrt {3}}{2}l^{2}$
*P*	Membrane rest length	*k*_*p*_ *L*_*p*_	2*π* *R*_*p*_	6*L*_*p*_
*A*	Target cell area	$k_{a}{L_{a}^{2}}$	$\pi {R_{a}^{2}}$	$\frac {3\sqrt {3}}{2}{L_{a}^{2}}$
*λ*_*p*_	Perimeter constraint	*λ*_*p*_	*λ*_*p*_	*λ*_*p*_
*λ*_*a*_	Area constraint	*λ*_*a*_	*λ*_*a*_	*λ*_*a*_
*l*	Basic length scale	*l*	*r* (radius)	*l*
*k*_*p*_	Perimeter scaling factor	$\frac {p}{l}$	2*π*	6
*k*_*a*_	Area scaling factor	$\frac {a}{l^{2}}$	*π*	$\frac {3\sqrt {3}}{2}$
*L*_*p*_	Membrane rest length, using basic length scale	$\frac {P}{k_{p}}$	$R_{p}=\frac {P}{2\pi }$	$\frac {P}{6}$
*L*_*a*_	Target cell area,using basic length scale	$\sqrt {\frac {A}{k_{a}}}$	$R_{a}=\sqrt {\frac {A}{\pi }}$	$\sqrt {\frac {2A}{3\sqrt {3}}}$
*E*	Energy function or Hamiltonian, using basic length scale	${Jk}_{p}l+\lambda _{p}(k_{p}l-k_{p}L_{p})^{2} +\lambda _{a}(k_{a}l^{2}-k_{a}{L_{a}^{2}})^{2}$	$2\pi rJ+\lambda _{p}(2\pi r-2\pi R_{p})^{2} +\lambda _{a}(\pi r^{2}-\pi {R_{a}^{2}})^{2}$	$6lJ+\lambda _{p}(6l-6L_{p})^{2} +\lambda _{a}(\frac {3\sqrt {3}}{2}l^{2}-\frac {3\sqrt {3}}{2}{L_{a}^{2}})^{2}$
$\frac {\partial E}{\partial l}$	Energy variation per length change	$k_{p}(\gamma -2\frac {k_{a}}{k_{p}}l\Pi)$	2*π*(*γ*−*r* *Π*)	$6(\gamma -\frac {\sqrt {3}}{2}l\Pi)$
*γ*	Enterfacial tension	*J*+2*k*_*p*_ *λ*_*p*_(*l*−*L*_*p*_)	*J*+4*π* *λ*_*p*_(*r*−*R*_*p*_)	*J*+12*λ*_*p*_(*l*−*L*_*p*_)
*Π*	Pressure	$-2k_{a}\lambda _{a}(l^{2}-{L_{a}^{2}})$	$-2\pi \lambda _{a}(r^{2}-{R_{a}^{2}})$	$-3\sqrt {3}\lambda _{a}(l^{2}-{L_{a}^{2}})$
*τ*	Length-independent component of interfacial tension	*J*−2*k*_*p*_ *λ*_*p*_ *L*_*p*_	*J*−4*π* *λ*_*p*_ *R*_*p*_	*J*−12*λ*_*p*_ *L*_*p*_
*ε*	*l* ^2^ at which $\frac {\partial ^{2}E}{\partial l^{2}}=0$	$\frac {{L_{a}^{2}}}{3}-\frac {{k_{p}^{2}}\lambda _{p}}{6{k_{a}^{2}}\lambda _{a}}$	$\frac {{R_{a}^{2}}}{3}-\frac {2\lambda _{p}}{3\lambda _{a}}$	$\frac {{L_{a}^{2}}}{3}-\frac {8\lambda _{p}}{9\lambda _{a}}$
*α*	$\left.\frac {\partial E}{\partial l}\right |_{l=\sqrt {\epsilon },\tau =0}$	$-8{k_{a}^{2}}\lambda _{a}\left (\frac {{L_{a}^{2}}}{3}-\frac {{k_{p}^{2}}\lambda _{p}}{6{k_{a}^{2}}\lambda _{a}}\right)^{\frac {3}{2}}$	$-8\pi ^{2}\lambda _{a}\left (\frac {{R_{a}^{2}}}{3}-\frac {2\lambda _{p}}{3\lambda _{a}}\right)^{\frac {3}{2}}$	$-54\lambda _{a}\left (\frac {{L_{a}^{2}}}{3}-\frac {8\lambda _{p}}{9\lambda _{a}}\right)^{\frac {3}{2}}$
*β*	Aggregate parameter	$4\frac {{k_{a}^{2}}\lambda _{a}}{k_{p}}$	2*π* *λ*_*a*_	$\frac {9\lambda _{a}}{2}$
*ζ*	Aggregate parameter	$\frac {64{k_{a}^{4}}{\lambda _{a}^{2}}}{{k_{p}^{2}}}\left (\frac {{k_{p}^{2}}\lambda _{p}}{6{k_{a}^{2}}\lambda _{a}}-\frac {{L_{a}^{2}}}{3}\right)^{3{\vphantom {\frac {1}{2}}}}$	$16\pi ^{2}{\lambda _{a}^{2}}\left (\frac {2\lambda _{p}}{3\lambda _{a}}-\frac {{R_{a}^{2}}}{3}\right)^{3}$	$81{\lambda _{a}^{2}}\left (\frac {8\lambda _{p}}{9\lambda _{a}}-\frac {{L_{a}^{2}}}{3}\right)^{3}$
Bifurcation 1 (*γ*(*l* ^∗^)=0)	Transition from negative to positive interfacial tension at equilibrium	*τ*=−2*k*_*p*_ *λ*_*p*_ *L*_*a*_	*τ*=−4*π* *λ*_*p*_ *R*_*a*_	*τ*=−12*λ*_*p*_ *L*_*a*_
Bifurcation 2 (pseudo-transcritical)	Transition of *l* ^∗^=0 from unstable to stable	*τ*=0	*τ*=0	*τ*=0
Bifurcation 3 (fold)	Transition from 2 to 0 non-trivial equilibria	$\tau =\frac {8{k_{a}^{2}}\lambda _{a}}{k_{p}}\epsilon ^{\frac {3}{2}}$	$\tau =4\pi \lambda _{a}\epsilon ^{\frac {3}{2}}$	$\tau =9\lambda _{a}\epsilon ^{\frac {3}{2}}$
$\overline {\Lambda }$	Normalised tension,as used in [25]	$\frac {J}{k_{a}^{\frac {3}{2}}\lambda _{a}{L_{a}^{3}}}$	$\frac {J}{\pi ^{\frac {3}{2}}\lambda _{a}{R_{a}^{3}}}$	$\frac {2\sqrt {2}J}{9\sqrt [4]{3}\lambda _{a}{L_{a}^{3}}}$
$\overline {\Gamma }$	Normalised contractility,as used in [25]	$\frac {\lambda _{p}}{k_{a}\lambda _{a}{L_{a}^{2}}}$	$\frac {\lambda _{p}}{\pi \lambda _{a}{R_{a}^{2}}}$	$\frac {2\lambda _{p}}{3\sqrt {3}\lambda _{a}{L_{a}^{2}}}$
Bifurcation 1 (*γ*(*l* ^∗^)=0)	Transition from negative to positive interfacial tension at equilibrium	$\overline {\Gamma }=-\frac {\sqrt {k_{a}}}{2k_{p}}\overline {\Lambda }$	$\overline {\Gamma }=-\frac {1}{4\sqrt {\pi }}\overline {\Lambda }$	$\overline {\Gamma }=-\frac {1}{4\sqrt {2}\sqrt [4]{3}}\overline {\Lambda }$
Bifurcation 2 (pseudo-transcritical)	Transition of *l* ^∗^=0 from unstable to stable	$\overline {\Lambda }=0$	$\overline {\Lambda }=0$	$\overline {\Lambda }=0$
Bifurcation 3 (fold)	Transition from 2 to 0non-trivial equilibria	$\overline {\Gamma }=\frac {4k_{a}-3\left (k_{a}k_{p}\overline {\Lambda }\right)^{\frac {2}{3}}}{2{k_{p}^{2}}}$	$\overline {\Gamma }=\frac {4\pi -3\left (2\pi ^{2}\overline {\Lambda }\right)^{\frac {2}{3}}}{8\pi ^{2}}$	$\overline {\Gamma }=\frac {2-3\sqrt [6]{3}\left (\overline {\Lambda }\right)^{\frac {2}{3}}}{8\sqrt {3}}$

### 3D cell packing

In densely-packed 3D tissues, cells take up the form of rhombic dodecahedrons (equivalent to the hexagonal shape in 2D tissues), which is the most efficient space-filling packing as it requires the smallest amount of surface area per volume. Again we first derive a general solution for any cell shape, introducing a surface scaling factor, $k_{s}=\frac {s}{l^{2}}$, and a volume scaling factor $k_{v}=\frac {v}{l^{3}}$. In Appendix E we find that after introducing such a generalization for cell shape, the bifurcation lines we found previously for the spherical shape (Eq. 27) become

(54a) $$\begin{array}{*{20}l} \nu &= -\frac{9{k_{v}^{2}}\lambda_{v}{L_{v}^{2}}}{2{k_{s}^{2}}\lambda_{s}}\,, \end{array} $$

(54b) $$\begin{array}{*{20}l} \nu &= 0\,, \end{array} $$

(54c) $$\begin{array}{*{20}l} \nu &= f(\mu)\left(\mu-f(\mu)\right)\,, \end{array} $$

where $\nu =\frac {\left (J-2k_{s}\lambda _{s}{L_{s}^{2}}\right)9{k_{v}^{2}}\lambda _{v}}{4{k_{s}^{3}}{\lambda _{s}^{2}}}=\frac {\tau }{12a\phi ^{2}}$, $\mu =\frac {27{k_{v}^{3}}\lambda _{v}^{\frac {3}{2}}{L_{v}^{3}}}{8{k_{s}^{3}}\lambda _{s}^{\frac {3}{2}}}=\frac {\psi }{\phi ^{\frac {3}{2}}}$, and $f(\mu)=\sinh \left (\frac {1}{3}\operatorname {arcsinh}\left (\mu \right)\right)$.

Again, we find that the bifurcation diagram does not qualitatively change due to this generalization of cell shape, and that the specific shape of individual cells only modifies the precise parameter values at which the bifurcations occur. Given that for the rhombic dodecahedron $k_{s}=8\sqrt {2}$ and $k_{v}=\frac {16}{3\sqrt {3}}$, the analytical expectation of the behaviour of 3D densely-packed tissue becomes defined by the two aggregate parameters

(55a) $$\begin{array}{*{20}l} \nu & =\frac{\left(J-16\sqrt{2}\lambda_{s}{L_{s}^{2}}\right)\lambda_{v}}{48\sqrt{2}{\lambda_{s}^{2}}}\,, \end{array} $$

(55b) $$\begin{array}{*{20}l} \mu & =\frac{{L_{v}^{3}}\lambda_{v}^{\frac{3}{2}}}{6\sqrt{6}\lambda_{s}^{\frac{3}{2}}}\,, \end{array} $$

with the bifurcation lines located at

(56a) $$\begin{array}{*{20}l} \nu &= -\frac{\lambda_{v}{L_{v}^{2}}}{3\lambda_{s}}\,, \end{array} $$

(56b) $$\begin{array}{*{20}l} \nu &= 0\,, \end{array} $$

(56c) $$\begin{array}{*{20}l} \nu &= f(\mu)(\mu-f(\mu))\,. \end{array} $$

Again, when the analytical results are compared to CPM simulations of a cell tissue, we find a very close correspondence between them. Figure 9B shows 3D CPM simulations of a small tissue for each of the four possible regions. It shows the individual cells within a cluster that is not in direct contact with the medium. As for the 2D case, in region I the cells take up an irregular shape, fulfilling both the surface area and the volume requirement. In region II, the cell shape is very regular, indeed very closely corresponding to a rhombic dodecahedron. In region III, the initial size of the individual cells determines whether the cells disappear or not, while in region IV all cells eventually disappear. The boundaries between those regions of different qualitative behaviour, as observed in the 3D CPM simulations, again closely match the analytical predictions (data not shown).

## Discussion

Our analytical results, in combination with computer simulations, have elucidated how the biophysical parameters of CSM models can be translated into qualitative cell dynamics. Some observations and underlying assumptions made during this process merit further discussion. To start with, in region I of the cellular dynamics, the observed behaviour is driven by a negative interfacial tension. However, it is questionable whether such a parameter regime is biologically and physically reasonable; the main issue being that negative interfacial tension implies the intermingling of neighbouring cells. This phenomenon is not observed in low resolution cell shape CSM models, which often use, for example, a single line element for the whole interface between two cells (e.g. [25]), effectively inhibiting cells from intermingling. However, in the CPM the cell’s interface is resolved at much higher detail, clearly exposing the intermingling between cells (see Figure 11C1). From a biological perspective, such dynamics might be considered pathological and unrealistic, and one should be weary of using such a dynamical regime to capture normal biological processes.

On the other range of the spectrum, it is also worth discussing whether it is reasonable to assume that cells can completely disappear (i.e. shrink to zero) due to their cell surface mechanics. One could argue that the elastic description of area and perimeter conservation becomes unreasonable when deviations become large, and that while water can freely enter and leave the cell, ultimately the dry cellular mass should prevent full disappearance. Nevertheless, experimental biological studies have been showing pressure-driven apoptosis [48,49], which has been used as a possible interpretation for vanishing cells in the CPM [50]. Another explanation has been proposed by Marinari et al. [16], in a combined experimental and modelling study of the *Drosophila* notum. They showed that in a 2D CSM description of the notum an increased pressure resulted in the local disappearance of cells, which was then attributed to the process of delamination, when cells leave the epithelial plane and enter the (non-described) underlying tissue.

Given that the most reasonable dynamics are found for (a large range of) well-balanced parameter choices, it will be important for most theoretical studies which are based upon CSM concepts — including the CPM — to apply the insights derived from our study: (i) to choose parameters sensibly, (ii) to ensure model behaviour matches experimental data, and (iii) to prevent potential modelling artefacts.

Although the analysis presented here is relevant for the large class of CSM models that use the same underlying building blocks, there is obviously a wide range of different model descriptions in use to capture cell dynamics. These alternative models cannot always be easily encapsulated within the same theoretical framework. For instance, other common strategies to describe the biophysics of cells is using the so-called subcellular elements method [51,52] or by modelling of cellular material as a continuous medium taking stress and strain tensors explicitly into account [53]. These modelling strategies grant a finer description of forces, not just at the membrane, as with CSM formalisms, but also within the cell. Although this level of detail is important when studying the cell rheology, quite often the simplifications defining CSM models make them better suited for morphodynamical simulations, where part of the individual cell detail may be exchanged for the feasibility of many-cell simulations.

Finally, we argue that the proposal suggested by Ouchi et al. [22] — to use negative instead of positive J-values (adhesion energy constant) to solve the apparent mismatch (regarding the relationship between adhesion and cell motility) between models and experiments — fails to reconcile experiments and simulations. Using both theory and simulations, we have shown here that this limitation is not particular to the CPM, but rather present in any modelling framework that describes the strength of adhesion through an effective lower tension.

## Conclusions

The analytical results presented here have numerous practical applications. First, they allow researchers to determine the expected dynamics due to interactions between volume conservation, adhesion and cortical tension in 2D and 3D CSM models. Table 4 presents a hands-on guide to be used as a protocol for determining the dynamics of any CSM study. Additionally, this study has shown how the CPM formalism fits within the realm of CSM models, and how biophysically quantitative simulations should be performed within this formalism. Table 5 provides a guide for the usage of the CPM in such a quantitative fashion. Furthermore, our analytical results directly predict the (change in) dynamics, given specific (modifications in) cellular properties, which can be used when linking simulations to biophysical experiments and predicting the experimental outcome. Finally, we have shown that the amount of possible dynamics is much more limited than the parameter space involved originally suggested. This implies that there is no unique combination of volume conservation, adhesion and cortical tension that leads to a given dynamical behaviour. One consequence is that it is impossible to derive from a single set of experimental observations a unique set of specific parameters that capture those dynamics. Using the analysis presented here, however, it becomes possible to specifically define the set of combinations of cellular properties that could explain the observed behaviour. It enables the pinpointing of the critical relationship between cell surface mechanics and tissue dynamics, which are not immediately clear from computational studies alone.

**Table 4:** **For the perplexed: how to determine the expected dynamics in any CSM model**

Step 1.	Write the Hamiltonian or energy function in the standard form as used in this paper (i.e. *E*=*J* *p*+*λ*_*p*_(*p*−*P*)^2^+*λ*_*a*_(*a*−*A*)^2^ for 2D; *E*=*J* *s*+*λ*_*s*_(*s*−*S*)^2^+*λ*_*v*_(*v*−*V*)^2^ for 3D).
Step 2.	When using CPM, first the effective values of *J*, and *P* and *λ*_*p*_ in 2D, or *S* and *λ*_*s*_ in 3D, have to be determined, or inversely the correct values of $J_{_{\mathit {CPM}}}$ etc. have to be assigned. This is done by calculating the perimeter scaling factor *ξ*, which depends on the neighbourhood radius, in its turn depending on the neighbourhood level. The value of *ξ* can be obtained either by using the theoretical formulae Eq. 29, Eq. 33, for 2D and 3D respectively, in the case that the neighbourhood is sufficiently large, or by using the numerical estimates given in Table 2, for small neighbourhoods that can present significant deviations from those theoretical values. Transformations between the effective and CPM parameter values are then given by Eq. 31, Eq. 32 for 2D, and Eq. 34 for 3D. Note that because of the way interfacial tension is implemented in the CPM, $J=\xi J_{_{\mathit {CPM}}}$ for cell-medium interfaces and $\frac {\xi }{2}J_{_{\mathit {CPM}}}$ for cell-cell interfaces.
Step 3.	Calculate *k*_*p*_, *k*_*a*_ (for 2D) cq. *k*_*s*_, *k*_*v*_ (for 3D) for the cell shape of interest. The values for typical shapes in 2D and 3D, namely circle and hexagon, cq. sphere and rhombic dodecahedron, are given in Table 3 and Table 6.
Step 4.	Calculate *τ* and *ε* (for 2D), *ν* and *μ* (for 3D when *λ*_*s*_≠0), or *ν* ^′^ and *μ* ^′^ (for 3D when *λ*_*s*_=0), using the formulae given in Table 3 (for 2D) and Table 6 (for 3D).
Step 5.	The expected behaviour of a single cell or tissue is given by Figure 3; the bifurcation lines which form the transitions between the different zones are given in Table 3 (for 2D) and Table 6 (for 3D).

**Table 5:** **For the perplexed: how to correctly rescale a CPM model**

***Rescale spatial resolution***	When the spatial resolution of a CPM model is *k*-fold increased, the required changes in the standard set of kinetic parameters are given in Eq. 38 and Eq. 39.
***Resizing neighbourhood***	When the neighbourhood used in a simulation is changed, the value of $J_{_{\mathit {CPM}}}\phantom {\dot {i}\!}$, and $P_{_{\mathit {CPM}}}\phantom {\dot {i}\!}$ and $\lambda _{p\,{}_{\mathit {CPM}}}\phantom {\dot {i}\!}$ in 2D, or $S_{_{\mathit {CPM}}}\phantom {\dot {i}\!}$ and $\lambda _{s\,{}_{\mathit {CPM}}}\phantom {\dot {i}\!}$ in 3D, have to be modified, such that the effective values remain the same (Eq. 31, Eq. 32, Eq. 34). This can be achieved by setting $J_{_{\mathit {CPM}}}'=\frac {\xi _{\textit {old}}}{\xi _{\textit {new}}}J_{_{\mathit {CPM}}}\phantom {\dot {i}\!}$, where *ξ*_*old*_ and *ξ*_*new*_ are the perimeter scaling factor before and after resizing the neighbourhood, respectively. Likewise, $P_{_{\mathit {CPM}}}'=\frac {\xi _{\textit {new}}}{\xi _{\textit {old}}}P_{_{\mathit {CPM}}}\phantom {\dot {i}\!}$, $\lambda _{p\,{}_{\mathit {CPM}}}'=\frac {\xi _{\textit {old}}^{2}}{\xi _{\textit {new}}^{2}}\lambda _{p\,{}_{\mathit {CPM}}}\phantom {\dot {i}\!}$, $S_{_{\mathit {CPM}}}'=\frac {\xi _{\textit {new}}}{\xi _{\textit {old}}}S_{_{\mathit {CPM}}}\phantom {\dot {i}\!}$, and $\lambda _{s\,{}_{\mathit {CPM}}}'=\frac {\xi _{\textit {old}}^{2}}{\xi _{\textit {new}}^{2}}\lambda _{s\,{}_{\mathit {CPM}}}\phantom {\dot {i}\!}$. Details on calculating *ξ* are in Step 2 of Table 4.
***Concurrent rescaling of J and P***	It is possible to concurrently change all *J* values (for example from all being positive to all being negative) in a CPM, or in fact any CSM simulation, without causing *any* change in the dynamics of the model (Figure 6), by means of well-chosen shifts in the membrane rest lengths of all cells (*P*_*σ*_ and *S*_*σ*_ in 2D and 3D, respectively). The required shifts in the rest lengths are given in Eq. 47 (for 2D) and Eq. 49 (for 3D). In contrast, it is not possible to change only a subset of the *J* values without causing changes in dynamics. Specifically, in such a case, it is still possible to keep for a specific configuration the weighted mean adhesion-driven interfacial tension constant (using Eq. 46 for 2D and Eq. 48 for 3D), but those weighted means are expected to change over time (for example due to cell sorting), generating an imbalance and hence a change of dynamics on the long run.

## Appendix

## A 3D Spherical Cell

### Positive interfacial tension at the equilibrium radii

We can express the derivative of *E* with respect to *r* in terms of the interfacial tension *γ* and the pressure *Π*

(57a) $$\begin{array}{*{20}l} \frac{\partial E}{\partial r} & =J\frac{\partial s}{\partial r}+2\lambda_{s}\frac{\partial s}{\partial r}\left(s-S\right)+2\lambda_{v}\frac{\partial v}{\partial r}\left(v-V\right)\,, \end{array} $$

(57b) $$\begin{array}{*{20}l} & =\frac{\partial s}{\partial r}\left(J+8\pi\lambda_{s}\left(r^{2}-R_{s}\right)\right)+\frac{8\pi}{3}\lambda_{v}\frac{\partial v}{\partial r}\left(r^{3}-{R_{v}^{3}}\right)\,, \end{array} $$

(57c) $$\begin{array}{*{20}l} & =8\pi r\gamma+\frac{8\pi}{3}\lambda_{v}4\pi r^{2}\left(r^{3}-{R_{v}^{3}}\right)\,, \end{array} $$

(57d) $$\begin{array}{*{20}l} & =4\pi r\left(2\gamma+\frac{8\pi}{3}r\lambda_{v}\left(r^{3}-{R_{v}^{3}}\right)\right)\,, \end{array} $$

(57e) $$\begin{array}{*{20}l} & =4\pi r\left(2\gamma-r\Pi\right)\,. \end{array} $$

At equilibrium *r*^∗^, *γ*(*r*^∗^) is positive when

(58a) $$\begin{array}{*{20}l} \tau+8\pi\lambda_{s}r^{*2} & >0\,, \end{array} $$

(58b) $$\begin{array}{*{20}l} \tau>0\vee r^{*} & >\sqrt{\frac{-\tau}{8\pi\lambda_{s}}}\,. \end{array} $$

Given that at equilibrium

(59) $$ \left.\frac{\partial E}{\partial r}\right|_{r=r^{*}}=4\pi r^{*}\left(2\gamma\left(r^{*}\right)-r^{*}\Pi\left(r^{*}\right)\right)=0\,,  $$

this yields

(60a) $$\begin{array}{*{20}l} \gamma\left(r^{*}\right) & >0\,, \end{array} $$

(60b) $$\begin{array}{*{20}l} r^{*}\Pi\left(r^{*}\right) & >0\,, \end{array} $$

(60c) $$\begin{array}{*{20}l} 0<r^{*} & <R_{v}\,. \end{array} $$

Combined, Eq. 58 and Eq. 60 give the condition

(61) $$ \tau>-8\pi\lambda_{s}{R_{v}^{2}}\,,  $$

which is very comparable to the condition expressed in Eq. 14b of the 2D case.

### Equilibrium radii and their stability

The derivative of *E* with respect to the radius of the cell is

(62a) $$\begin{array}{*{20}l} \frac{\partial E}{\partial r} & =8\pi Jr+16\pi\lambda_{s}r\left(4\pi r^{2}-4\pi {R_{s}^{2}}\right)\\ &\quad+8\pi\lambda_{v}r^{2}\left(\frac{4}{3}\pi r^{3}-\frac{4}{3}\pi {R_{v}^{3}}\right)\,, \end{array} $$

(62b) $$\begin{array}{*{20}l} &=8\pi\left(Jr+8\pi\lambda_{s}r\left(r^{2}-{R_{s}^{2}}\right)+\frac{4}{3}\pi\lambda_{v}r^{2}\left(r^{3}-{R_{v}^{3}}\right)\right)\,, \end{array} $$

(62c) $$\begin{array}{*{20}l} &=8\pi\left(\frac{4}{3}\pi\lambda_{v}r^{5}+8\pi\lambda_{s}r^{3}-\frac{4}{3}\pi\lambda_{v}{R_{v}^{3}}r^{2}\right.\\ &\quad+\left.(J-8\pi\lambda_{s}{R_{s}^{2}})r{\vphantom{\frac{1}{2}}}\right)\,, \end{array} $$

(62d) $$\begin{array}{*{20}l} & =8\pi\left(ar^{5}+br^{3}-cr^{2}+\tau r\right)\,, \end{array} $$

where $\tau =J-8\pi \lambda _{s}{R_{s}^{2}}$, which can be both positive or negative; $a=\frac {4}{3}\pi \lambda _{v}$, strictly positive; and *b*=8*πλ*_*s*_ and $c=\frac {4}{3}\pi \lambda _{v}{R_{v}^{3}}$, both strictly non-negative.

We can use Descartes’ rule of signs to determine the highest number of equilibria larger than zero (remember that *r*^∗^<0 is nonsensical). When *τ*<0, there is only one sign change, limiting the number of equilibria larger than zero to at most one, while when *τ*>0, two sign changes limit it to at most two.

Further insights can be obtained by looking at the second derivative of the energy *E*,

(63) $$ \frac{\partial^{2}E}{\partial r^{2}}=8\pi\left(5ar^{4}+3br^{2}-2cr+\tau\right)\,.  $$

The slope of the function $\frac {\partial E}{\partial r}$ at the equilibrium *r*^∗^=0 is 8*πτ*, implying that this equilibrium is always stable for *τ*>0 (positive slope), and always unstable for *τ*<0 (negative slope). (Recall that the force on the cell membrane is positive in the direction of the energy gradient steepest descent.)

Moreover, because $\left.\frac {\partial E}{\partial r}\right |_{r=0}=0$ and $\left.\frac {\partial E}{\partial r}\right |_{r\rightarrow \infty }=\infty $, a negative slope implies an odd number of equilibria larger than zero, while a positive slope implies an even number. Combined with the insights derived from Descartes’ rule of signs, we can therefore conclude that when *τ*<0, there is at least and at most one equilibrium for *r*>0, i.e. there is always one single equilibrium. Also, this equilibrium has to be stable, since the slope at the equilibrium has to be positive. When *τ*>0, there can be either no equilibria, or two positive equilibria. When there are two equilibria, the lower one will be the unstable and the higher one the stable.

The next step is to determine those parameter regimes for *τ*>0 without and with two positive equilibria. Both regimes are separated by a bifurcation for which we want to find a parametrisation. We only need to analyse the subset of our parameter space for which *τ*>0. The bifurcation is a fold bifurcation, and the transition from two to no equilibria implies that, at the bifurcation point, $\left.\frac {\partial E}{\partial r}\right |_{r=r^{*}}=0$ and $\left.\frac {\partial ^{2}E}{\partial r^{2}}\right |_{r=r^{*}}=0$. Moreover, we know that *r*^∗^>0. Hence, at the bifurcation point

(64a) $$\begin{array}{*{20}l} ar^{5}+br^{3}-cr^{2}+\tau r & =0 \quad \wedge\qquad \hspace*{68pt}5ar^{4}+3br^{2}-2cr+\tau =0\,, \end{array} $$

(64b) $$\begin{array}{*{20}l} ar^{5}+br^{3}-cr^{2}+\tau r & =0 \quad \wedge \quad \hspace*{69.5pt} 5ar^{5}+3br^{3}-2cr^{2}+\tau r =0\,, \end{array} $$

(64c) $$\begin{array}{*{20}l} 3ar^{5}+3br^{3}-3cr^{2}+3\tau r & =0 \quad \wedge \hspace*{79pt} 5ar^{5}+3br^{3}-2cr^{2}+\tau r =0\,, \end{array} $$

(64d) $$\begin{array}{*{20}l} 4ar^{5}+2br^{3}-cr^{2} & =0 \quad \wedge \hspace*{74pt} 8ar^{5}+6br^{3}-5cr^{2}+4\tau r =0\,, \end{array} $$

(64e) $$\begin{array}{*{20}l} r^{2}\left(4ar^{3}+2br-c\right) & =0 \quad \wedge \quad 2r^{2}\left(4ar^{3}+2br-c\right)+2br^{3}-3cr^{2}+4\tau r =0\,, \end{array} $$

(64f) $$\begin{array}{*{20}l} r^{2}\left(4ar^{3}+2br-c\right) & =0 \quad \wedge \hspace*{99pt} r\left(2br^{2}-3cr+4\tau\right) =0\,, \end{array} $$

(64g) $$\begin{array}{*{20}l} 4ar^{3}+2br-c & =0 \quad \wedge \hspace*{112pt} 2br^{2}-3cr+4\tau =0\,. \end{array} $$

This reduces, at the bifurcation point, the fifth and fourth order equations into a cubic and a quadratic equation, respectively. The cubic equation is a reduced cubic equation (see Eq. 64g, left-hand side). If we define $\phi =\frac {b}{6a}=\frac {\lambda _{s}}{\lambda _{v}}$ and $\psi =\frac {c}{8a}=\frac {{R_{v}^{3}}}{8}$, the cubic equation can be written as

(65) $$ r^{3}+3\phi r-2\psi=0\,.  $$

Given that *b* is strictly non-negative and *a* strictly positive, *ϕ*≥0. We first study when *ϕ*>0. The only positive solution of this cubic equation is then given by [54,55]

(66a) $$\begin{array}{*{20}l} r^{*}&=2\sqrt{\phi}\sinh\left(\frac{1}{3}\text{arcsinh}\left(\frac{\psi}{\phi^{\frac{3}{2}}}\right)\right)\,, \end{array} $$

(66b) $$\begin{array}{*{20}l} r^{*} & =2\sqrt{\phi}\sinh\left(\frac{1}{3}\text{arcsinh}\left(\mu\right)\right)\,, \end{array} $$

(66c) $$\begin{array}{*{20}l} r^{*} & =2\sqrt{\phi}f(\mu)\,, \end{array} $$

where $\mu =\frac {\psi }{\phi ^{\frac {3}{2}}}=\frac {{R_{v}^{3}}}{8\left (\frac {\lambda _{s}}{\lambda _{v}}\right)^{\frac {3}{2}}}$ and $f(\mu)=\sinh \left (\frac {1}{3}\operatorname {arcsinh}\left (\mu \right)\right)$. From the quadratic equation (Eq. 64g, right-hand side) one can derive the value of *τ*,

(67a) $$\begin{array}{*{20}l} \tau & =\frac{1}{4}r\left(3c-2br\right)\,, \end{array} $$

(67b) $$\begin{array}{*{20}l} \tau & =\frac{1}{4}r\left(24a\frac{3c}{24a}-24a\frac{1}{2}\frac{4b}{24a}r\right)\,, \end{array} $$

(67c) $$\begin{array}{*{20}l} \tau & =6ar\left(\psi-\frac{1}{2}\phi r\right)\,, \end{array} $$

(67d) $$\begin{array}{*{20}l} \tau & =12a\sqrt{\phi}f(\mu)\left(\psi-\phi^{\frac{3}{2}}f(\mu)\right)\,, \end{array} $$

(67e) $$\begin{array}{*{20}l} \tau & =12a\phi^{2}f(\mu)\left(\frac{\psi}{\phi^{\frac{3}{2}}}-f(\mu)\right)\,, \end{array} $$

(67f) $$\begin{array}{*{20}l} \frac{\tau}{12a\phi^{2}} & =f(\mu)\left(\mu-f(\mu)\right)\,, \end{array} $$

(67g) $$\begin{array}{*{20}l} \nu & =f(\mu)\left(\mu-f(\mu)\right)\,, \end{array} $$

where $\nu =\frac {\tau }{12a\phi ^{2}}=\frac {\left (J-8\pi \lambda _{s}{R_{s}^{2}}\right)\lambda _{v}}{16{\pi \lambda _{s}^{2}}}$. Because both *a* and *ϕ* are positive, it still holds that when *ν*<0, there is only one, stable positive equilibrium, while when *ν*>0, the bifurcation line *ν*=*f*(*μ*)(*μ*−*f*(*μ*)) separates the region with two positive from the region without equilibria. Since *f*(*μ*)<*μ* for any positive *μ*, the line lies in the first quadrant of the (*μ*,*ν*)-plane. There are two equilibria as long as *ν*<*f*(*μ*)(*μ*−*f*(*μ*)), or *ν*−*f*(*μ*)(*μ*−*f*(*μ*))<0. The different regimes are shown in Figure 3B, and the bifurcation lines are given in Table 6.

**Table 6:** **Parameters used in the 3D analysis and their meaning**

**Parameter**	**Meaning**	**General**	**Sphere**	**Rhombic dodecahedron**
*E*	Energy function or Hamiltonian	*J* *s*+*λ*_*s*_(*s*−*S*)^2^+*λ*_*v*_(*v*−*V*)^2^		
*J*	Edhesion energy (per contact length)	*J*	*J*	*J*
*s*	Cell surface	*k*_*s*_ *l* ^2^	4*π* *r* ^2^	$8\sqrt {2}l^{2}$
*v*	Cell volume	*k*_*v*_ *l* ^3^	$\frac {4}{3}\pi r^{3}$	$\frac {16}{3\sqrt {3}}l^{3}$
*S*	Rest surface area	$k_{s}{L_{s}^{2}}$	$4\pi {R_{s}^{2}}$	$8\sqrt {2}{L_{s}^{2}}$
*V*	Ttarget cell volume	$k_{v}{L_{v}^{3}}$	$\frac {4}{3}\pi {R_{v}^{3}}$	$\frac {16}{3\sqrt {3}}{L_{v}^{3}}$
*λ*_*s*_	Surface constraint	*λ*_*s*_	*λ*_*s*_	*λ*_*s*_
*λ*_*v*_	Volume constraint	*λ*_*v*_	*λ*_*v*_	*λ*_*v*_
*l*	Basic length scale	*l*	*r* (radius)	*l*
*k*_*s*_	Surface scaling factor	$\frac {s}{l^{2}}$	4*π*	$8\sqrt {2}$
*k*_*v*_	Volume scaling factor	$\frac {v}{l^{3}}$	$\frac {4}{3}\pi $	$\frac {16}{3\sqrt {3}}$
*L*_*s*_	Rest surface area, using basic length scale	$\sqrt {\frac {S}{k_{s}}}$	$R_{s}=\frac {1}{2}\sqrt {\frac {S}{\pi }}$	$\sqrt {\frac {S}{8\sqrt {2}}}$
*L*_*v*_	Target cell volume,using basic length scale	$\sqrt [3]{\frac {V}{k_{v}}}$	$R_{v}=\sqrt [3]{\frac {3V}{4\pi }}$	$\sqrt [3]{\frac {3\sqrt {3}V}{16}}$
*E*	Energy function or Hamiltonian, using basic length scale	${Jk}_{s}l^{2}+\lambda _{s}(k_{s}l^{2}-k_{s}{L_{s}^{2}})^{2} +\lambda _{v}(k_{v}l^{3}-k_{v}{L_{v}^{3}})^{2}$	$4\pi Jr^{2}+\lambda _{s}(4\pi r^{2}-4\pi {R_{s}^{2}})^{2} +\lambda _{v}(\frac {4}{3}\pi r^{3}-\frac {4}{3}\pi {R_{v}^{3}})^{2}$	$8\sqrt {2}Jl^{2}$ $+\lambda _{s}(8\sqrt {2}l^{2}-8\sqrt {2}{L_{s}^{2}})^{2} +\lambda _{v}(\frac {16}{3\sqrt {3}}l^{3}-\frac {16}{3\sqrt {3}}{L_{v}^{3}})^{2}$
$\frac {\partial E}{\partial l}$	Energy variation per length change	$2k_{s}l\left (\gamma -\frac {3k_{v}}{2k_{s}}l\Pi \right)$	4*π* *r*(2*γ*−*r* *Π*)	$16\sqrt {2}l\left (\gamma -\frac {l}{\sqrt {6}}\Pi \right)$
*γ*	Interfacial tension	$J+2k_{s}\lambda _{s}(l^{2}-{L_{s}^{2}})$	$J+8\pi \lambda _{s}(r^{2}-{R_{s}^{2}})$	$J+16\sqrt {2}\lambda _{s}(l^{2}-{L_{s}^{2}})$
*Π*	Pressure	$-2k_{v}\lambda _{v}(l^{3}-{L_{v}^{3}})$	$-\frac {8}{3}\pi \lambda _{v}(r^{3}-{R_{v}^{3}})$	$-\frac {32}{3\sqrt {3}}\lambda _{v}(l^{3}-{L_{v}^{3}})$
$\frac {\partial E}{\partial l}$	Energy variation per length change, full expansion	2*k*_*s*_(*a* *l* ^5^+*b* *l* ^3^−*c* *l* ^2^+*τ* *l*)	8*π*(*a* *r* ^5^+*b* *r* ^3^−*c* *r* ^2^+*τ* *r*)	$16\sqrt {2}\left (al^{5}+bl^{3}-cl^{2}+\tau l\right)$
*a*	Aggregate parameterin $\frac {\partial E}{\partial l}$ equation	$\frac {3{k_{v}^{2}}\lambda _{v}}{k_{s}}$	$\frac {4}{3}\pi \lambda _{v}$	$\frac {16}{9}\sqrt {2}\lambda _{v}$
*b*	Aggregate parameterin $\frac {\partial E}{\partial l}$ equation	2*k*_*s*_ *λ*_*s*_	8*π* *λ*_*s*_	$16\sqrt {2}\lambda _{s}$
*c*	Aggregate parameterin $\frac {\partial E}{\partial l}$ equation	$\frac {3{k_{v}^{2}}\lambda _{v}{L_{v}^{3}}}{k_{s}}$	$\frac {4}{3}\pi \lambda _{v}{R_{v}^{3}}$	$\frac {16}{9}\sqrt {2}\lambda _{v}{L_{v}^{3}}$
*τ*	Length-independent component of interfacial tension	$J-2k_{s}\lambda _{s}{L_{s}^{2}}$	$J-8\pi \lambda _{s}{R_{s}^{2}}$	$J-16\sqrt {2}\lambda _{s}{L_{s}^{2}}$
*ϕ*	$\frac {b}{6a}$	$\frac {{k_{s}^{2}}\lambda _{s}}{9{k_{v}^{2}}\lambda _{v}}$	$\frac {\lambda _{s}}{\lambda _{v}}$	$\frac {3\lambda _{s}}{2\lambda _{v}}$
*ψ*	$\frac {c}{8a}$	$\frac {{L_{v}^{3}}}{8}$	$\frac {{R_{v}^{3}}}{8}$	$\frac {{L_{v}^{3}}}{8}$
*ν* (when *ϕ*>0)	$\frac {\tau }{12a\phi ^{2}}$	$\frac {\left (J-2k_{s}\lambda _{s}{L_{s}^{2}}\right)9{k_{v}^{2}}\lambda _{v}}{4{k_{s}^{3}}{\lambda _{s}^{2}}}$	$\frac {\left (J-8\pi \lambda _{s}{R_{s}^{2}}\right)\lambda _{v}}{16{\pi \lambda _{s}^{2}}}$	$\frac {\left (J-16\sqrt {2}\lambda _{s}{L_{s}^{2}}\right)\lambda _{v}}{48\sqrt {2}{\lambda _{s}^{2}}}$
*μ*(when *ϕ*>0)	$\frac {\psi }{\phi ^{\frac {3}{2}}}$	$\frac {27{k_{v}^{3}}\lambda _{v}^{\frac {3}{2}}{L_{v}^{3}}}{8{k_{s}^{3}}\lambda _{s}^{\frac {3}{2}}}$	$\frac {{R_{v}^{3}}\lambda _{v}^{\frac {3}{2}}}{8\lambda _{s}^{\frac {3}{2}}}$	$\frac {{L_{v}^{3}}\lambda _{v}^{\frac {3}{2}}}{6\sqrt {6}\lambda _{s}^{\frac {3}{2}}}$
*ν* ^′^(when *ϕ*=0)	$\frac {\tau }{12a}$	$\frac {{Jk}_{s}}{36{k_{v}^{2}}\lambda _{v}{\vphantom {\frac {1}{2}}}}$	$\frac {J}{16\pi \lambda _{v}}$	$\frac {3J}{64\sqrt {2}\lambda _{v}}$
*μ* ^′^(when *ϕ*=0)	*ψ*	$\frac {{L_{v}^{3}}}{8}$	$\frac {{R_{v}^{3}}}{8}$	$\frac {{L_{v}^{3}}}{8}$
Bifurcation 1 (*γ*(*l* ^∗^)=0)	Transition from negative to positive interfacial tension at equilibrium	$\nu =-\frac {9{k_{v}^{2}}\lambda _{v}{L_{v}^{2}}}{2{k_{s}^{2}}\lambda _{s}}$	$\nu =-\frac {\lambda _{v}{R_{v}^{2}}}{2\lambda _{s}}$	$\nu =-\frac {\lambda _{v}{L_{v}^{2}}}{3\lambda _{s}}$
		*ν* ^′^=0	*ν* ^′^=0	*ν* ^′^=0
Bifurcation 2 (pseudo-transcritical)	Transition of *l* ^∗^=0 from unstable to stable	*ν*=0	*ν*=0	*ν*=0
		*ν* ^′^=0	*ν* ^′^=0	*ν* ^′^=0
Bifurcation 3 (fold)	Transition from 2 to 0 non-trivial equilibria	*ν*=*f*(*μ*)(*μ*−*f*(*μ*)), where $f(\mu)=\sinh \left (\frac {1}{3}\text {arcsinh} \left (\mu \right)\right)$	*ν*=*f*(*μ*)(*μ*−*f*(*μ*))	*ν*=*f*(*μ*)(*μ*−*f*(*μ*))
		$\nu '=\frac {\mu '^{\frac {4}{3}}}{2^{\frac {2}{3}}}$	$\nu '=\frac {\mu '^{\frac {4}{3}}}{2^{\frac {2}{3}}}$	$\nu '=\frac {\mu '^{\frac {4}{3}}}{2^{\frac {2}{3}}}$

Alternatively, for the specific case when *ϕ*=0 (meaning that *λ*_*s*_=0), the solution of Eq. 65 is simply

(68) $$ r^{*}=\sqrt[3]{2\psi}\,.  $$

Moreover, in this case the quadratic equation (Eq. 64g, right-hand side) reduces to a linear equation, allowing again to derive the value of *τ* at which the bifurcation occurs,

(69a) $$\begin{array}{*{20}l} \tau & =\frac{3}{4}cr\,, \end{array} $$

(69b) $$\begin{array}{*{20}l} \tau & =6a\psi\sqrt[3]{2\psi}\,, \end{array} $$

(69c) $$\begin{array}{*{20}l} \frac{\tau}{12a} & =\frac{\psi^{\frac{4}{3}}}{2^{\frac{2}{3}}}\,, \end{array} $$

(69d) $$\begin{array}{*{20}l} \nu' & =\frac{\mu^{'\frac{4}{3}}}{2^{\frac{2}{3}}}\,, \end{array} $$

where $\nu '=\frac {\tau }{12a}=\frac {J}{16\pi \lambda _{v}}$ (taking into account that the specific case of *ϕ*=0 only occurs when *λ*_*s*_=0, and hence *τ* is equal to *J*) and $\mu '=\psi =\frac {{R_{v}^{3}}}{8}$. When *ϕ*=0, due to the absence of a surface constraint, the *γ*(*r*^∗^)=0 bifurcation takes place at *ν*^′^=0. This is the same condition as for the pseudo-transcritical bifurcation, while the bifurcation line $\nu '=\frac {\mu '^{\frac {4}{3}}}{2^{\frac {2}{3}}}$ separates the region with two positive equilibria from the region without equilibria, again located in the first quadrant of the (*μ*^′^,*ν*^′^)-plane.

## B Cell packing: derivation of the equations for the hexagonal cell

In order to derive the equations for a hexagonal cell shape, we write the equations for a general family of shapes, for which the perimeter and area can be parametrised as *p*=*k*_*p*_*l* and *a*=*k*_*a*_*l*^2^, respectively, with *l* being a basic length scale. Substituting *a*=*k*_*a*_*l*^2^, *p*=*k*_*p*_*l*, $A=k_{a}{L_{a}^{2}}$ and *P*=*k*_*p*_*L*_*p*_ into the general equation *E*(*p*,*a*)=*Jp*+*λ*_*p*_(*p*−*P*)^2^+*λ*_*a*_(*a*−*A*)^2^ yields

(70) $$\begin{array}{*{20}l} &E\left(\left.l\right|J,\lambda_{p},L_{p},\lambda_{a},L_{a},k_{p},k_{a}\right)={Jk}_{p}l+\lambda_{p}\left(k_{p}l-k_{p}L_{p}\right)^{2}\\ &\quad+\lambda_{a}\left(k_{a}l^{2}-k_{a}{L_{a}^{2}}\right)^{2}\,. \end{array} $$

The interfacial tension *γ* and pressure *Π* are then given by

(71a) $$\begin{array}{*{20}l}  \gamma &= \ \ \frac{\partial E}{\partial p} \ \hspace*{1pt} =J+2k_{p}\lambda_{p}(l-L_{p})=\tau+2k_{p}\lambda_{p}l\,, \end{array} $$

(71b) $$\begin{array}{*{20}l} \Pi &= -\frac{\partial E}{\partial a} =-2k_{a}\lambda_{a}(l^{2}-{L_{a}^{2}})\,, \end{array} $$

where *τ*=*J*−2*λ*_*p*_*k*_*p*_*L*_*p*_, with the same meaning as before. From Eq. 71a, it follows that the length *l* at which the interfacial tension is zero, is given by

(72) $$ l_{\gamma=0}=-\frac{\tau}{2k_{p}\lambda_{p}}\,.  $$

The derivative of the energy function *E*(*l*) is easily obtained by differentiation of Eq. 70:

(73a) $$\begin{array}{*{20}l} \frac{\partial E}{\partial l} &={Jk}_{p}+2\lambda_{p}{k_{p}^{2}}\left(l-L_{p}\right)+2\lambda_{a}{k_{a}^{2}}\left(l^{2}-{L_{a}^{2}}\right)2l\,, \end{array} $$

(73b) $$\begin{array}{*{20}l} &=k_{p}\left(J+2\lambda_{p}k_{p}\left(l-L_{p}\right)\right)+2k_{a}l\left(2k_{a}\lambda_{a}\left(l^{2}-{L_{a}^{2}}\right)\right)\,, \end{array} $$

(73c) $$\begin{array}{*{20}l} &=k_{p}\gamma-2k_{a}l\Pi\,. \end{array} $$

Thus, as before, at equilibrium zero tension implies zero pressure. The first bifurcation line can therefore be determined by combining Eq. 71b and Eq. 72:

(74a) $$\begin{array}{*{20}l} \Pi & =0\,, \end{array} $$

(74b) $$\begin{array}{*{20}l} l & =L_{a}\,, \end{array} $$

(74c) $$\begin{array}{*{20}l} \tau & =-2k_{p}\lambda_{p}L_{a}\,. \end{array} $$

Using a same reasoning as for the circular shape (see Eq. 11b, Eq. 12b and text below it), it follows that the interfacial tension at any non-trivial equilibrium is always positive when *τ*>−2*λ*_*p*_*k*_*p*_*L*_*a*_, and negative otherwise.

The stability of the trivial equilibrium *l*^∗^=0 can be determined as follows. Evaluating the derivative of the energy function (Eq. 73c) at *l*^∗^=0 gives

(75) $$ \left.\frac{\partial E}{\partial l}\right|_{l^{*}=0}=k_{p}\gamma(0)=\tau\,.  $$

Consequently, as for the circular cell shape, the condition *τ*=0 defines the second bifurcation line, with *τ*<0 implying an unstable trivial equilibrium, and *τ*>0 a stable one. To find the third bifurcation line requires analysis of the second derivative of the energy function, given by

(76a) $$\begin{array}{*{20}l} \frac{\partial^{2}E}{\partial l^{2}} & =k_{p}\gamma'-2k_{a}\Pi-2k_{a}l\Pi'\,, \end{array} $$

(76b) $$\begin{array}{*{20}l} &=k_{p}(2\lambda_{p}k_{p})-2k_{a}2\lambda_{a}k_{a}({L_{a}^{2}}-l^{2})+8{k_{a}^{2}}\lambda_{a}l^{2}\,, \end{array} $$

(76c) $$\begin{array}{*{20}l} &=2{k_{p}^{2}}\lambda_{p}-4{k_{a}^{2}}\lambda_{a}({L_{a}^{2}}-l^{2})+8{k_{a}^{2}}\lambda_{a}l^{2}\,, \end{array} $$

(76d) $$\begin{array}{*{20}l} &=k_{p}\left(2k_{p}\lambda_{p}-\frac{4{k_{a}^{2}}\lambda_{a}{L_{a}^{2}}}{k_{p}}+12\frac{{k_{a}^{2}}\lambda_{a}}{k_{p}}l^{2}\right)\,, \end{array} $$

(76e) $$\begin{array}{*{20}l} &=3k_{p}\beta\left(l^{2}-\epsilon\right)\,, \end{array} $$

where $\beta =4\frac {{k_{a}^{2}}\lambda _{a}}{k_{p}}$ and $\epsilon =\frac {{L_{a}^{2}}}{3}-\frac {{k_{p}^{2}}\lambda _{p}}{6{k_{a}^{2}}\lambda _{a}}$ are aggregate parameters. We have pointed out before that the third bifurcation line can be found when the inflection point of the energy function coincides with the equilibrium cell length *l*^∗^ (see Figure 2). We therefore first find the cell length *l* at the inflection point:

(77a) $$\begin{array}{*{20}l} \frac{\partial^{2}E}{\partial l^{2}} & =0\,, \end{array} $$

(77b) $$\begin{array}{*{20}l} l^{2}-\epsilon & =0\,, \end{array} $$

(77c) $$\begin{array}{*{20}l} l & =\sqrt{\epsilon}\,. \end{array} $$

Thus, the aggregate parameter *ε* can be interpreted as the inflection point of the energy function squared, with negative values of *ε* implying that there is no inflection point. We next rewrite the first derivative, now using those aggregate parameters:

(78a) $$\begin{array}{*{20}l} \frac{\partial E}{\partial l} & =&k_{p}\gamma-2k_{a}l\Pi\,, \end{array} $$

(78b) $$\begin{array}{*{20}l} &=k_{p}(\gamma-2\frac{k_{a}}{k_{p}}l\Pi)\,, \end{array} $$

(78c) $$\begin{array}{*{20}l} &=k_{p}(\tau+2\lambda_{p}k_{p}l-2\frac{k_{a}}{k_{p}}l\Pi)\,, \end{array} $$

(78d) $$\begin{array}{*{20}l} &=k_{p}\left(\tau+4\frac{{k_{a}^{2}}\lambda_{a}}{k_{p}}l\left(l^{2}-{L_{a}^{2}}+\frac{\lambda_{p}{k_{p}^{2}}}{2{k_{a}^{2}}\lambda_{a}}\right)\right)\,, \end{array} $$

(78e) $$\begin{array}{*{20}l} &=k_{p}\left(\tau+\beta l\left(l^{2}-3\epsilon\right)\right)\,. \end{array} $$

The third bifurcation line is then given by:

(79a) $$\begin{array}{*{20}l} \left.\frac{\partial E}{\partial l}\right|_{l=\sqrt{\epsilon}} & =0\,, \end{array} $$

(79b) $$\begin{array}{*{20}l} k_{p}\left(\tau-2\beta\epsilon^{\frac{3}{2}}\right) & =0\,, \end{array} $$

(79c) $$\begin{array}{*{20}l} \tau & =2\beta\epsilon^{\frac{3}{2}}\,. \end{array} $$

In conclusion, two equilibria with positive, real values requires that $0<\tau <2\beta \epsilon ^{\frac {3}{2}}\,$.

To obtain convenient expressions of the equilibria themselves, we introduce again the aggregate parameter *α*, the slope at the inflection point given that *τ*=0:

(80a) $$\begin{array}{*{20}l} \left.\frac{\partial E}{\partial l}\right|_{l=\sqrt{\epsilon},\tau=0} & =k_{p}\left(\beta\sqrt{\epsilon}(\epsilon-3\epsilon)\right)\,, \end{array} $$

(80b) $$\begin{array}{*{20}l} \alpha & =-2k_{p}\beta\epsilon^{\frac{3}{2}}\,, \end{array} $$

(80c) $$\begin{array}{*{20}l} \alpha & =-8{k_{a}^{2}}\lambda_{a}\epsilon^{\frac{3}{2}}\,. \end{array} $$

After introducing *α*, the first derivative can be written as

(81) $$ \frac{\partial E}{\partial l}=k_{p}\left(\tau-\frac{\alpha}{2k_{p}\epsilon^{\frac{3}{2}}}l(l^{2}-3\epsilon)\right)\,.  $$

When *ε*>0, $\tau <-\frac {\alpha }{k_{p}}$, the stable, non-trivial positive equilibrium is then given by (note that in this case *α* is a negative real number)

(82a) $$\begin{array}{@{}rcl@{}} l_{1} =2\sqrt{\epsilon}\cos\left(\frac{1}{3}\arccos\left(\frac{\tau k_{p}}{\alpha}\right)\right)\,, \end{array} $$

rewritten when $\tau <\frac {\alpha }{k_{p}}$ into

(82b) $$\begin{array}{@{}rcl@{}} l_{1,alt} =2\sqrt{\epsilon}\cosh\left(\frac{1}{3}\text{arccosh}\left(\frac{\tau k_{p}}{\alpha}\right)\right)\,, \end{array} $$

to prevent trigonometry involving complex numbers. When *ε*>0, $0<\tau <-\frac {\alpha }{k_{p}}$, there is a second non-trivial equilibrium, which is non-stable,

(82c) $$\begin{array}{@{}rcl@{}} l_{2} =2\sqrt{\epsilon}\sin\left(\frac{1}{3}\arcsin\left(-\frac{\tau k_{p}}{\alpha}\right)\right)\,, \end{array} $$

and when *ε*<0, *τ*<0 there is one non-trivial positive equilibrium, which is stable. Because in the latter case *α* is a positive imaginary number, we again use the aggregate parameter $\zeta =-\left (\frac {\alpha }{k_{p}}\right)^{2}$, which is positive and real when *ε*<0. The equilibrium radius is then given by

(82d) $$ l_{3}=\frac{\sqrt{-\epsilon}}{\sqrt[6]{\zeta}}\left(\sqrt[3]{-\tau+\sqrt{\tau^{2}+\zeta}}-\sqrt[3]{\tau+\sqrt{\tau^{2}+\zeta}}\right)\,.  $$

For the hexagon, the perimeter *p* and area *a* can be parametrised as *p*=6*l* and $a=\frac {3\sqrt {3}}{2}l^{2}$. Thus, *k*_*p*_=6 and $k_{a}=\frac {3\sqrt {3}}{2}$. The first bifurcation then becomes *τ*=−12*λ*_*p*_*L*_*a*_. The second bifurcation is given by *τ*=0 and the third by $\tau =2\beta \epsilon ^{\frac {3}{2}}$, with *k*_*p*_=6 and $k_{a}=\frac {3\sqrt {3}}{2}$ substituted into *τ*, *β* and *ε*. Table 3 gives an overview of all parameters used and their meaning, for the general cell shape as well as specifically for the circle and hexagon.

## C Additional parameter information regarding the CPM simulations

*Figure 5*

In the single cell CPM simulations, a 6th level neighbourhood was used (and hence a correction factor *ξ*=18 for scaling corrections, see Table 2). For the first simulation, *k*=1; *J*=80,000 ($J_{_{\mathit {CPM}}}=4,444\phantom {\dot {i}\!}$); *A*=7,854 (*R*_*a*_=1,250); *P*=0 ($P_{_{\mathit {CPM}}}=0\phantom {\dot {i}\!}$); *λ*_*a*_=2; and *λ*_*p*_=25 ($\lambda _{p\,{}_{\mathit {CPM}}}=0.07716\phantom {\dot {i}\!}$) (for switching between actual and CPM parameters, see Eq. 31, Eq. 32). For all other simulations the parameters were rescaled using Eq. 38.

In the cell sorting CPM simulations, a Moore neighbourhood was used. For the first simulation, *k*=1; *J*_*G*,*G*_=*J*_*Y*,*Y*_=*J*_*B*,*B*_=400, *J*_*G*,*M*_=600, *J*_*Y*,*M*_=1,200, *J*_*B*,*M*_=1,800, *J*_*G*,*Y*_=*J*_*Y*,*B*_=800, *J*_*G*,*B*_=1,400; *A*=30; *P*=100; *λ*_*a*_=1,000; *λ*_*p*_=20; and *T*=600. (Here, only the CPM parameters are given, which can be translated into CSM values using Eq. 31.) In the *k*≠1 simulations the parameters were rescaled using Eq. 38.

*Figure 7*

The initial cell radii used to illustrate the dynamics in the different regions were as follows: For Region I (C2), *r*={10, 50, 70, 150}; for Region II (D2), *r*={10, 50, 70, 150}; for Region III (E2), *r*={10, 20, 30, 36, 50, 70, 115, 150}; and for Region IV (F2) *r*={50, 70, 100, 150}. All simulations used a 6th level neighbourhood and *A*=31,416 (*R*_*a*_=100); *P*=2,000 ($P_{_{\mathit {CPM}}}=36,000\phantom {\dot {i}\!}$); *λ*_*a*_=0.0625; *λ*_*p*_=25 ($\lambda _{p\,{}_{\mathit {CPM}}}=0.07716\phantom {\dot {i}\!}$); and *T*=20,000. The *J*-value was modified to set *τ*; in regions I–IV, *J*=0 ($J_{_{\mathit {CPM}}}=0\phantom {\dot {i}\!}$), *J*=80,000 ($J_{_{\mathit {CPM}}}=4,444\phantom {\dot {i}\!}$), *J*=200,000 ($J_{_{\mathit {CPM}}}=11,111\phantom {\dot {i}\!}$), and *J*=300,000 ($J_{_{\mathit {CPM}}}=16,667\phantom {\dot {i}\!}$), respectively.

*Figure 11*

As the initial condition, all cells were positioned within a region of 300×300 lattice points, except for (F1), which utilized 100×100 lattice points. All simulations used a 6th level neighbourhood and *A*=31,416 (*L*_*a*_=110, *R*_*a*_=100); *P*=2,000 ($P_{_{\mathit {CPM}}}=36,000\phantom {\dot {i}\!}$); *λ*_*a*_=0.0625; *λ*_*p*_=25 ($\lambda _{p\,{}_{\mathit {CPM}}}=0.07716\phantom {\dot {i}\!}$); and *T*=20,000. The *J*-values were modified to set *τ*, in which $J_{_{\mathit {CPM}}}=J_{C,M}=2J_{C,C}\phantom {\dot {i}\!}$. In regions I–IV, *J*=0 ($J_{_{\mathit {CPM}}}=0\phantom {\dot {i}\!}$), *J*=80,000 ($J_{_{\mathit {CPM}}}=4,444\phantom {\dot {i}\!}$), *J*=200,000 ($J_{_{\mathit {CPM}}}=11,111\phantom {\dot {i}\!}$), and *J*=300,000 ($J_{_{\mathit {CPM}}}=16,667\phantom {\dot {i}\!}$), respectively.

## D Notation correspondence to Farhadifar et al. (2007)

In the paper of Farhadifar et al. [25] a same CSM energy description was used to simulate epithelial cell packing. In order to make a straightforward comparison, we introduce a mapping to their notation. In their study, *L*_*p*_=0, and consequently *τ*=*J*. The parameter equivalences are

(83a) $$\begin{array}{*{20}l} \tau(=J) \rightarrow & \Lambda\,, \end{array} $$

(83b) $$\begin{array}{*{20}l} \lambda_{p} \rightarrow & \Gamma\,, \end{array} $$

(83c) $$\begin{array}{*{20}l} \lambda_{a} \rightarrow & K\,, \end{array} $$

(83d) $$\begin{array}{*{20}l} k_{a}{L_{a}^{2}} \rightarrow & A^{0}\,, \end{array} $$

(83e) $$\begin{array}{*{20}l} L_{p} \rightarrow & 0\,. \end{array} $$

The authors also define the composite parameters normalised tension $\overline {\Lambda }$ and normalised contractility $\overline {\Gamma }$, which are used to depict their biophysical parameter space. These are defined as:

(84a) $$\begin{array}{*{20}l} \overline{\Lambda} &=\frac{\Lambda}{K\left(A^{0}\right)^{\frac{3}{2}}}\rightarrow\frac{J}{k_{a}^{\frac{3}{2}}\lambda_{a}{L_{a}^{3}}}\,, \end{array} $$

(84b) $$\begin{array}{*{20}l} \overline{\Gamma} &=\frac{\Gamma}{KA^{0}}\rightarrow\frac{\lambda_{p}}{k_{a}\lambda_{a}L_{a}^{2}}\,. \end{array} $$

$\overline {\Gamma }$ can be expressed as a function of $\overline {\Lambda }$:

(85) $$ \overline{\Gamma}=\frac{\Gamma\sqrt{A^{0}}}{\Lambda}\overline{\Lambda}\,.  $$

The bifurcation line defining negative or positive interfacial tension is given by *τ*=−2*k*_*p*_*λ*_*p*_*L*_*a*_. Given that *L*_*p*_= 0, both *τ*=*J* and *Λ*=*J*, and thus this condition can be expressed in their notation as

(86) $$ \overline{\Gamma}=-\frac{\sqrt{k_{a}}}{2k_{p}}\overline{\Lambda}\,.  $$

For the hexagon this yields

(87) $$ \overline{\Gamma}=-\frac{1}{4\sqrt{2}\sqrt[4]{3}}\overline{\Lambda}\,,  $$

which was also derived in [30]. Because *L*_*p*_=0, the bifurcation line at *τ*=0 is simply given by $\overline {\Lambda }=0$. Using the derivation in the previous section, the third bifurcation line is given by

(88) $$ \overline{\Gamma}=\frac{4k_{a}-3\left(k_{a}k_{p}\overline{\Lambda}\right)^{\frac{2}{3}}}{2{k_{p}^{2}}}\,,  $$

which for the hexagon yields

(89) $$ \overline{\Gamma}=\frac{2-3\sqrt[6]{3}\left(\overline{\Lambda}\right)^{\frac{2}{3}}}{8\sqrt{3}}\,.  $$

The bifurcation line defined by Eq. 89 has the $\overline {\Gamma }$-intercept at $\overline {\Gamma }=\frac {2}{8\sqrt {3}}$ and $\overline {\Lambda }$-intercept at $\overline {\Lambda }=\frac {2\sqrt {2}}{3\cdot 3^{\frac {3}{4}}}$.

## E 3D cell packing: derivation of the equations for the rhombic dodecahedronal cell

After introducing the surface and volume scaling factors *s*=*k*_*s*_*l*^2^ and *v*=*k*_*v*_*l*^3^, the general equation yields

(90) $$\begin{array}{@{}rcl@{}} E={Jk}_{s}l^{2}+\lambda_{s}(k_{s}l^{2}-k_{s}{L_{s}^{2}})^{2}+\lambda_{v}(k_{v}l^{3}-k_{v}{L_{v}^{3}})^{2}\,. \end{array} $$

The interfacial tension and pressure are then given by

(91a) $$\begin{array}{*{20}l} \gamma &= \ \ \frac{\partial E}{\partial s} \ \hspace*{1pt} = J+2k_{s}\lambda_{s}(l^{2}-{L_{s}^{2}})= \tau+2k_{s}\lambda_{s}l^{2}\,, \end{array} $$

(91b) $$\begin{array}{*{20}l} \Pi &= -\frac{\partial E}{\partial v} = -2k_{v}\lambda_{v}(l^{3}-{L_{v}^{3}})\,, \end{array} $$

where $\tau =J-2k_{s}\lambda _{s}{L_{s}^{2}}$. The first step is to find the cell size *l* for which the interfacial tension is zero:

(92a) $$\begin{array}{*{20}l} \gamma & =0=\tau+2k_{s}\lambda_{s}l^{2}\,, \end{array} $$

(92b) $$\begin{array}{*{20}l} l & =\sqrt{-\frac{\tau}{2k_{s}\lambda_{s}}}\,. \end{array} $$

Because zero tension implies zero pressure, the first bifurcation line is given by

(93a) $$\begin{array}{*{20}l} \Pi & =&0\,, \end{array} $$

(93b) $$\begin{array}{*{20}l} l & =&L_{v}\,, \end{array} $$

(93c) $$\begin{array}{*{20}l} \tau & =&-2k_{s}\lambda_{s}{L_{v}^{2}}\,. \end{array} $$

The second bifurcation line is, as usual, given by *τ*=0. For the third bifurcation line, we take the derivative of the energy function

(94a) $$\begin{array}{*{20}l} \frac{\partial E}{\partial l} & =2k_{s}l\left(\gamma-\frac{3k_{v}}{2k_{s}}l\Pi\right)\,, \end{array} $$

(94b) $$\begin{array}{*{20}l} &=2{Jk}_{s}l+4\lambda_{s}k_{s}l\left(k_{s}l^{2}-k_{s}{L_{s}^{2}}\right)\\ &\quad+6\lambda_{v}k_{v}l^{2}\left(k_{v}l^{3}-k_{v}{L_{v}^{3}}\right)\,, \end{array} $$

(94c) $$\begin{array}{*{20}l} &=2k_{s}\left(\frac{3\lambda_{v}{k_{v}^{2}}}{k_{s}}l^{5}+2\lambda_{s}k_{s}l^{3}-\frac{3\lambda_{v}{k_{v}^{2}}{L_{v}^{3}}}{k_{s}}l^{2}\right.\\ &\quad+\left.{\vphantom{\frac{9}{9}}}\left(J-2k_{s}\lambda_{s}{L_{s}^{2}}\right)l\right)\,, \end{array} $$

(94d) $$\begin{array}{*{20}l} &=2k_{s}\left(al^{5}+bl^{3}-cl^{2}+\tau l\right)\,. \end{array} $$

In Appendix A we already showed that the roots of this equation correspond to the solutions of the cubic equation *l*^3^+3*ϕl*−2*ψ*=0, where $\phi =\frac {b}{6a}$ and $\psi =\frac {c}{8a}$. The specific value of those aggregate parameters, however, becomes more complex for the general case:

(95a) $$\begin{array}{*{20}l} \phi & =\frac{b}{6a} =\frac{2\lambda_{s}{k_{s}^{2}}}{18\lambda_{v}{k_{v}^{2}}} =\frac{{k_{s}^{2}}}{9{k_{v}^{2}}}\frac{\lambda_{s}}{\lambda_{v}}\,, \end{array} $$

(95b) $$\begin{array}{*{20}l} \psi & =\frac{c}{8a} =\frac{\frac{3{k_{v}^{2}}}{k_{s}}\lambda_{v}{L_{v}^{3}}k_{s}}{24\lambda_{v}{k_{v}^{2}}} =\frac{{L_{v}^{3}}}{8}\,. \end{array} $$

The further derivation is equivalent to the spherical case, with the second and third bifurcation located at *ν*=0 and *ν*=*f*(*μ*)(*μ*−*f*(*μ*)), respectively, where $f(\mu)=\sinh \left (\frac {1}{3}\operatorname {arcsinh}\left (\mu \right)\right)$, when *ϕ*>0; and at *ν*^′^=0 and $\nu '=\frac {\mu '^{\frac {4}{3}}}{2^{\frac {2}{3}}}$ when *ϕ*=0. Nevertheless, the aggregate parameters obtain a slightly different meaning:

(96a) $$\begin{array}{*{20}l} \nu &=\frac{\tau}{12a\phi^{2}} =\frac{\left(J-2k_{s}\lambda_{s}{L_{s}^{2}}\right)9{k_{v}^{2}}\lambda_{v}}{4{k_{s}^{3}}{\lambda_{s}^{2}}}\,, \end{array} $$

(96b) $$\begin{array}{*{20}l} \mu &= \ \ \ \frac{\psi}{\phi^{\frac{3}{2}}} \ \ \  =\frac{27{k_{v}^{3}}{L_{v}^{3}}\lambda_{v}^{\frac{3}{2}}}{8{k_{s}^{3}}\lambda_{s}^{\frac{3}{2}}}\,, \end{array} $$

(96c) $$\begin{array}{*{20}l} \nu' &= \ \ \ \frac{\tau}{12a} \ =\frac{{Jk}_{s}}{36{k_{v}^{2}}\lambda_{v}}\,, \end{array} $$

(96d) $$\begin{array}{*{20}l} \mu' &= \ \ \ \ \psi \ \ \ \ =\frac{{L_{v}^{3}}}{8}\,. \end{array} $$

Finally, to depict the bifurcation diagram (Figure 3B), the first bifurcation has to be expressed in *ν*:

(97a) $$\begin{array}{*{20}l} \tau & =-2k_{s}\lambda_{s}{L_{v}^{2}}\,, \end{array} $$

(97b) $$\begin{array}{*{20}l} \nu & =\frac{\tau}{12a\phi^{2}}=\frac{-2k_{s}\lambda_{s}{L_{v}^{2}}}{12a\phi^{2}}\,, \end{array} $$

(97c) $$\begin{array}{*{20}l} \nu & =\frac{-9{k_{v}^{2}}\lambda_{v}{L_{v}^{2}}}{2{k_{s}^{2}}\lambda_{s}}\,. \end{array} $$

When *ϕ*=0, the bifurcation is simply at *ν*^′^=0. Having derived the general equations, the specific equations for a rhombic dodecahedronal cell can be straightforwardly derived using $k_{s}=8\sqrt {2}$ and $k_{v}=\frac {16}{3\sqrt {3}}$, and are given in Table 6.

## Abbreviations

2D: 2-dimensional Euclidean space
3D: 3-dimensional Euclidean space
CPM: Cellular Potts model
CSM: Cell surface mechanics
DAH: Differential adhesion hypothesis
MCS: Monte Carlo step
